# Population genomic analyses of the chocolate tree, *Theobroma cacao* L., provide insights into its domestication process

**DOI:** 10.1038/s42003-018-0168-6

**Published:** 2018-10-16

**Authors:** Omar E. Cornejo, Muh-Ching Yee, Victor Dominguez, Mary Andrews, Alexandra Sockell, Erika Strandberg, Donald Livingstone, Conrad Stack, Alberto Romero, Pathmanathan Umaharan, Stefan Royaert, Nilesh R. Tawari, Pauline Ng, Osman Gutierrez, Wilbert Phillips, Keithanne Mockaitis, Carlos D. Bustamante, Juan C. Motamayor

**Affiliations:** 1 School of Biological Sciences, Washington State University, PO Box 644236, Heald Hall 429B, Pullman, Washington 99164 USA; 2 Department of Genetics, School of Medicine, Stanford University, 300 Pasteur Dr. Lane Bldg Room L331, Stanford, CA 94305 USA; 3 Stanford Functional Genomics Facility, Stanford, CA 94305 USA; 4 Department of Biology, Indiana University, 915 E. Third St, Bloomington, IN 47405 USA; 5 Biomedical Informatics Training Program, 1265 Welch Road, MSOB, X-215, MC 5479, Stanford, CA 94305-5479 USA; 6 Mars, Incorporated, 6885 Elm Street, McLean, VA 22101 USA; 7 United States Department of Agriculture-Agriculture Research Service, Subtropical Horticulture Research Station, 13601 Old Cutler Rd, Miami, FL 33158 USA; 8 Cocoa Research Centre, The University of the West Indies, St. Augustine, Trinidad and Tobago; 9 Computational and Systems Biology, Genome Institute of Singapore, 60 Biopolis Street, Genome, #02-01, Singapore, 138672 Singapore; 10 SHRS, USDS-ARS, 13601 Old Cutler Road, Miami, FL 33158 USA; 11 Programa de Mejoramiento de Cacao, CATIE, 7170 Turrialba, Costa Rica; 12 Pervasive Technology Institute, Indiana University, 2709 E. 10th St., Bloomington, IN 47408 USA

## Abstract

Domestication has had a strong impact on the development of modern societies. We sequenced 200 genomes of the chocolate plant *Theobroma cacao* L. to show for the first time to our knowledge that a single population, the Criollo population, underwent strong domestication ~3600 years ago (95% CI: 2481–13,806 years ago). We also show that during the process of domestication, there was strong selection for genes involved in the metabolism of the colored protectants anthocyanins and the stimulant theobromine, as well as disease resistance genes. Our analyses show that domesticated populations of *T. cacao* (Criollo) maintain a higher proportion of high-frequency deleterious mutations. We also show for the first time the negative consequences of the increased accumulation of deleterious mutations during domestication on the fitness of individuals (significant reduction in kilograms of beans per hectare per year as Criollo ancestry increases, as estimated from a GLM, *P* = 0.000425).

## Abstract (web summary)

Omar Cornejo et al. report a genomic analysis of 200 cacao plants (*Theobroma cacao* L.) representing more than 10 genetically distinct populations. They identify metabolic and disease resistance genes as contributing to the domestication of cacao and show that domesticated populations maintain a high proportion of deleterious mutations.

## Introduction

Organized-state societies were only possible after the development of agriculture, which involved the domestication of numerous plants and animals^1,2^. We are just starting to understand this process more from a genetic perspective, thanks to the expanding genomic technologies. Of particular interest is identifying the demographic scenario, timeline for domestication, and genomic consequences for species that have been critical in the development of societies^2–4^. Among all species, there is a special place in the general culture for the domestication of *Theobroma cacao* L, the plant from which chocolate is made. The chocolate tree has played a fundamental role in the development of Mesoamerican civilizations^5^ and has been a topic of research for over 100 years, but its domestication history has remained controversial^6–8^. While the domestication history of cacao has sparked the interest across diverse disciplines, our knowledge of the process is incomplete and often involves partial information focused on a few genetic groups, a few geographical regions, fragmented archeological information, or a limited number of genetic markers^8–12^. In this work, we report and analyze whole-genome variation of 200 *T. cacao* individuals (Supplementary Table 1) to investigate the evolutionary origin of Criollo, the cacao tree domesticated in Mesoamerica. We also examine the consequences of the domestication process in the genomic architecture of the accumulation of deleterious mutations along the genome, which in turn allowed us to understand critical limits of Criollo (domesticated) cacao productivity.

The process of domestication of cacao has sparked the interest of a diverse set of disciplines and yet our knowledge of the process is incomplete and often involves partial information focused either on only a few genetic groups, a few geographical regions, fragmented archeological information, or a limited number of genetic markers^8–12^. Current dogma suggests that cacao was introduced to Mesoamerica in Olmec times from the cacao varieties present in the Upper Amazon (Northern South America), the hotbed of diversity for the species^6,8^. Another line of evidence suggests that the route of domestication of the chocolate tree could have dispersed throughout the Amazon Basin along two routes: one leading north and another leading west^13^. According to this hypothesis, domestication of cacao would have occurred in South America and then spread to Central America and Mexico, carried during trade by native Americans^14^. Anthropological research supports the view of a domestication event occurring in Mesoamerica^6,9,12^. In addition, the continuous intermixing of farmed and wild cacao trees has likely continued to shape both gene pools in recent times^11,15,16^. Both the impact of ancient domestication processes and modern hybridization on the genetic variation in the species are largely unknown in *T. cacao*.

Traditional classifications of cacao have recognized the Criollo and Forastero groups or cultivars, and an additional hybrid of Criollo × Forastero called Trinitario^7^. Biological characteristics of these groups have been described. More detailed genetic analyses using microsatellite markers have uncovered a large number of genetic groups as well as a clear differentiation between the trees found in the Amazon Basin and the Criollo varieties found in Central America^10^. This work helped characterize the cacao germplasm into ten major genetically differentiated groups: Amelonado, Contamana, Criollo, Curaray, Guianna, Iquitos, Marañon, Nacional, Nanay, and Purús^10^. Additional analyses performed with microsatellites suggested that Criollo, the most likely representative of the cacao domesticated in Mesoamerica, is more closely related to trees from the Colombia–Ecuador border than trees from other South American groups^10^. Yet, there is a huge gap in information about the extent of genomic variation in the species, which makes it difficult to propose clear scenarios for the evolution of natural populations and the domestication of *T. cacao*. Although it is widely recognized that some of these ten populations have contributed in recent times to the genetic makeup of crops, the majority of them remain as wild populations^10^. The most closely related species to *T. cacao* is putatively *Theobroma grandiflorum*^17^, but the biological characteristics of the trees and the fruits are dramatically different to those found in *T. cacao*^18^.

In this work, we explore the extent of whole-genome variation in *T. cacao* L. and investigate the evolutionary origin of Criollo, the cacao tree domesticated in Mesoamerica. We provide a detailed analysis of the population genetic structure in the species and analyze the evolutionary history of the populations, with an emphasis on domestication and the process of selection during domestication. Finally, we show how the reproductive system in cacao and the relatively recent process of domestication have strongly influenced the accumulation of deleterious mutations in domesticated cacao, with measurable consequences on the fitness of the individuals. This last result is a strong validation of the cost of domestication hypothesis which proposes that the process of domestication of a species will result in the increase in number, frequency, or proportion of deleterious genetic variants, hindering genetic improvement of domesticated species^19,20^. We show not only that indeed, there is an increase in the higher-frequency deleterious variants, but also show that this increase is associated with a reduction of individual fitness in domesticated cacao.

## Results

### Genetic variation in *Theobroma cacao* L.

**Fig. 1:**
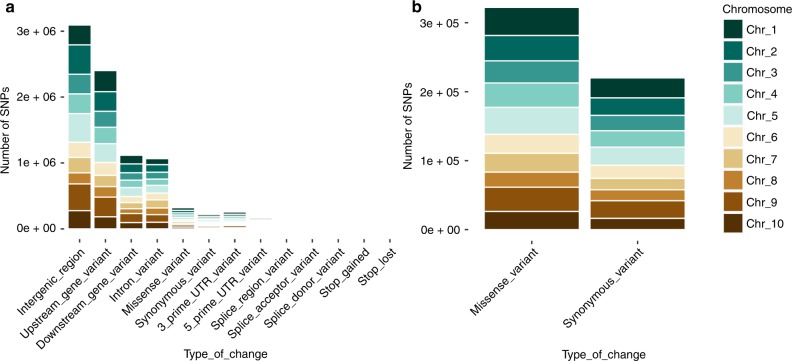
Genomic annotation of single-nucleotide polymorphism (SNPs) in *T. cacao*. **a** The number of SNPs categorized by functional impact in transcript variation per chromosome. **b** Details of the comparative number of synonymous and non-synonymous mutations

Resequencing of 200 accessions (see Supplementary Table 1 for details on accessions selected for the study) at high coverage (average coverage 22×) generated ~4.52 trillion base pairs. After aligning the reads to the cacao reference (Matina-v1.1^21^), we identified 7,412,507 single-nucleotide polymorphisms (SNPs). We found that cacao presents a high genetic variability of ~5 SNPs per kilobase per individual, similar to what has been observed in Arabidopsis^22,23^ (Supplementary Figure 1). Although the large majority of identified variants are noncoding, we identified 322,275 (4.35% of the total SNPs) missense variants and 220,043 (2.97% of the total SNPs) synonymous variants in 29,408 genes. We also identified 10,062 variants predicted to change the splice donor sites, which could be responsible for polymorphism, impacting the number of transcripts produced^21^. Among the potential changes that alter the length of the transcripts, we identified 8470 start losses (0.114% of total SNPs), 16,956 stop gains (0.229% of total SNPs), and 8588 stop losses (0.116% of total SNPs). Overall, this SNP dataset represents a new resource for cacao biology that we hope will accelerate breeding programs (for a catalog of SNPs contextualized with respect to gene annotation, see Supplementary Table 2 and Fig. 1).

### Population genetic structure and signature of domestication

**Fig. 2:**
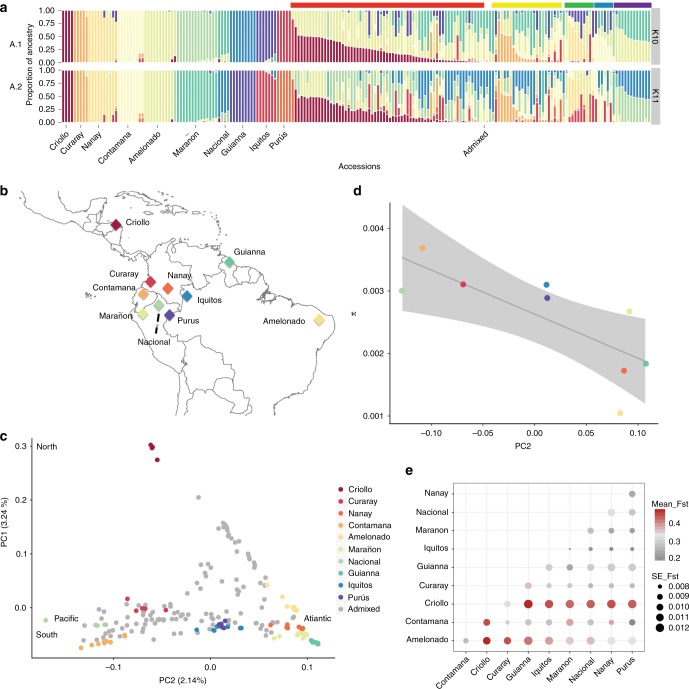
Population genetic structure in *T. cacao*. **a** The ten main genetic clusters can be recovered (A.1), although further structure (11 clusters) seems to be meaningful given that a considerable number of admixed individuals present the ancestry from a subset of Amelonado ancestry (A. 2). Color bars on top of the admixed individuals show our suggested grouping for the hybrids. **b** Map of Central and South America showing the median coordinate locations for the origin of samples from each population sampled in this work (with the exception of Admixed). **c** MDS showing a gradient of differentiation form the West to the East side of the Amazon (PC2) and a major separation of the Criollo group that corresponds to the Mesoamerican domesticated group (PC1). **d** Significant decay of genetic diversity (π) for the species along PC2 is supportive of the origin of the species being in the western side of the Amazon Basin (Criollo is excluded, model: π ∼ group + ε, *p* < 2E-16, r^2^ = 0.19). **e** All ten population genetic groups that have been described for the species are highly differentiated, with Criollo presenting a larger average F_ST_ when compared against all the other groups

Model-based clustering analysis using *ADMIXTURE*^24^ enabled us to identify ten genetic clusters, consistent with previous analyses^10^, and allowed us to correctly assign overall ancestry to previously uncharacterized accessions (Fig. 2a). We also present, for the first time, estimates of global ancestry for natural admixed individuals and man-made hybrids, revealing the relative contribution of the ten main populations to individual ancestry (Fig. 2a and Supplementary Figure 2). Our results show that there is an overrepresentation of genetic material from Amelonado, Criollo, and Nacional ancestry in the majority of admixed individuals. There is a concomitant underutilization of other genetic groups in current agronomist practice, which provides Blue Ocean opportunities for crop improvement (see Supplementary Information). Analyses of genetic diversity show significant differences in π across populations (Supplementary Table 3, *p* < 2e^-16^) and along the genomes within populations, a pattern that is consistent with what has been observed in other species (see Supplementary Information for more details and Supplementary Figures 4 and 5).

Further analysis of the population structure shows that the Criollo group is clearly differentiated from the rest of the genetic clusters along the first axis of a multidimensional scaling (MDS) analysis (Fig. 2b). The second component of the MDS analysis presents a gradient separating genetic clusters roughly from Pacific to Atlantic (bottom to top of the second component), consistent with a natural process of differentiation from the higher diversity groups on the Pacific side of the Amazonian Basin to those of lower diversity on the Atlantic side (See Fig. 2c, excluding domesticated Criollo, Y = 0.202 – 72.71 × , *p* = 0.02, Supplementary Table 4). It has been proposed that the center of origin of the species is located in the Western Amazon^7,25^. Our observation of the significant decline (*p* = 0.02 as per the model above) in genetic diversity from Pacific to Atlantic is consistent with the suggested center of origin for the species. The spread of admixed individuals in the MDS space is consistent with our admixture analysis in which individuals fall into two general categories: one which presents admixture between Criollo and Amelonado with a minor contribution from other groups and other hybrids presenting admixture along the Atlantic–Pacific gradient. There is a pattern of strong differentiation between all genetic clusters (Fig. 2d, *F*_ST_ values range between 0.16 and 0.65), with a larger differentiation between Criollo and any other group, consistent with either a scenario of strong drift during a recent process of domestication or an old diversification of the Criollo population from the rest of the genetic clusters. Given previous anthropological and genetic evidence^6,12,14^, it is more likely that this pattern is the result of domestication from a small pool of seeds that were used to create the Criollo group (drift), however, it is also possible that the strong divergence of Criollo from all the other groups is the result of a combination of both scenarios (diversification of the Criollo ancestral population and human-mediated genetic drift).

**Fig. 3:**
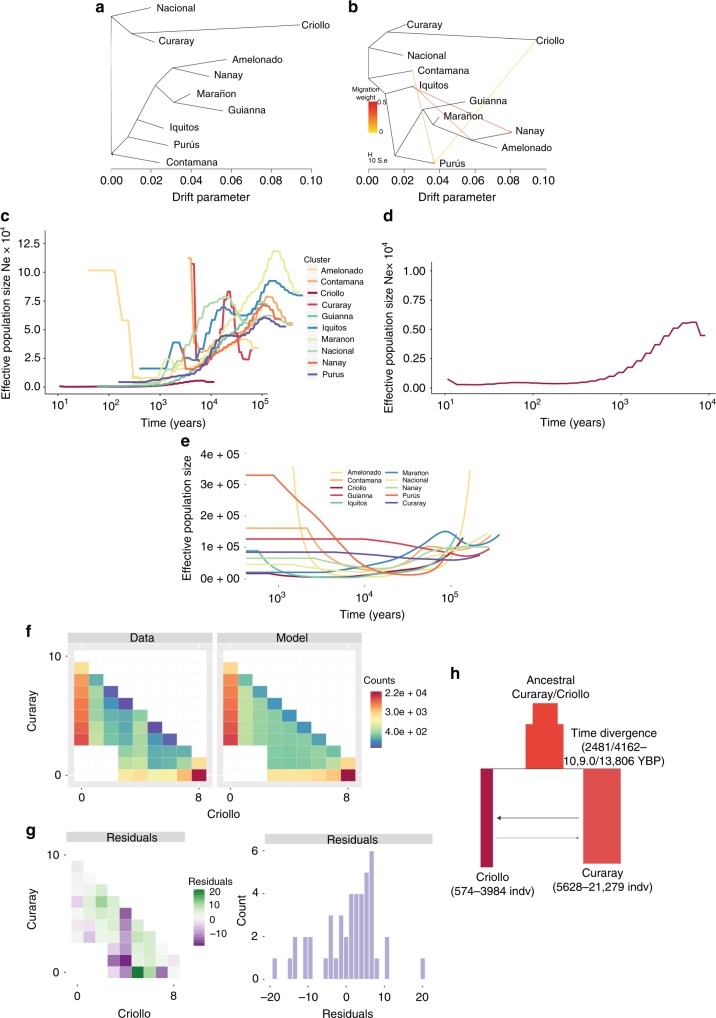
Population Demographics of *T. cacao*. **a** Maximum likelihood tree generated by *TreeMix* using intergenic regions of whole-genome sequencing data from individuals belonging to each one of the 10 main genetic groups. **b** Maximum likelihood tree allowing for admixture, as generated by *TreeMix*, showing some of the most significant ancestral contributions (migrations) from and to other groups. **c** Changes in effective population sizes over time, inferred under the coalescent with PSMC, for each on the 10 genetic groups in cacao. Each line represents the within-population median estimate, smoothed by fitting a cubic spline. **d** Detail of PSMC effective population size reconstruction for Criollo cacao, represented at a different scale to better represent the population decline. **e** Changes in effective population sizes over time, inferred under the coalescent with SMC + + , for each on the 10 genetic groups in cacao. Different color lines correspond to each population. A similar trend of historical population reduction (albeit different magnitudes) was observed with the two methods. **f** Observed two-dimensional site frequency spectrum (SFS, left panel) for the Criollo/Curaray population pair and expected SFS (right panel) under the inferred demographic model depicted in **g** The colors correspond to magnitudes (number of SNPs in each minor allele frequency bins). Anscombe residuals (difference between observed and expected) per frequency bin (left panel) and as an overall distribution (right panel). **h** Diagram for the proposed demographic model to explain Criollo/Curaray divergence, a model of isolation with migration. The time progresses from top to bottom and horizontal size of the boxes are relative to the relative effective population size. The estimated migration is relatively higher going from Curaray to Criollo, yet the scale of recombination estimated from the model is small

The hypothesis of a single event of domestication (along with genetic drift after transport from South to Central America) predicts that Criollo would show a higher differentiation to other groups than that observed between any other pairwise comparison of the populations. Our population structure analyses are consistent with this prediction (Fig. 2b, d). Our model-based analysis of population differentiation with TreeMix^26^ provides evidence that Criollo was the result of a single domestication event, undergoing extreme drift after separating from its most closely related population (represented as a longer branch in Fig. 3a, b, similar structure was obtained in a Neighbor-Joining analysis presented in Supplementary Figure 3). This analysis also shows that Criollo is most closely related to Curaray, suggesting that the origin of domesticated cacao was a subset of ancient Curaray germplasm^10^, a genetic cluster that has been described for the North of Ecuador and South of Colombia^10,27^. After exploring multiple models of differentiation with admixture, we found no evidence supporting subsequent contributions of any group to the domesticated Criollo, with the exception of a potential recent contribution of Purus to Criollo (Fig. 2b). However, we learned from this analysis that multiple instances of admixture have potentially occurred among multiple groups during their natural process of differentiation along the Amazon Basin (see Supplementary Information).

### Evolutionary genomics *Theobroma cacao* L.

In addition to admixture and population differentiation analyses, we investigated the demographic history of the ten genetic clusters to understand the natural demographic process that has characterized the species historically. Given the relatively small number of accessions per group, we performed analysis with pairwise sequentially Markovian coalescent (PSMC) model^28^ and smc++^29^, which allows evolutionary history inference by analyzing single diploid genomes. In general, the evolutionary history of *T. cacao* shows a common trend toward the reduction of population size/genetic diversity with time (Fig. 3c, d, and e). The median demographic history among accessions was used to show the overall trends for the evolutionary history of the groups. The process of reduction of effective population size started prior to the peopling of the Americans, which suggests that the overall reduction of genetic diversity in the species could be tied to environmental changes or historical changes in the distribution of pollinators and/or animals that are involved in the dispersion of the seeds during the Last Glacial Maximum. This result is consistent with recent studies suggesting that most groups of *Theobroma cacao* could have diversified during the last glaciation^27^. During the Last Glacial Maximum, it has been inferred that the Amazon had dry seasons that lasted twice as long as present day and rainfall could have dropped 25–35% from present day records and presented remarkably lower CO_2_ concentration in the atmosphere^30,31^. Pockets of increased humidity and more constant temperature during the year were constrained to the vicinity of major river basins and the development of refugia^30,31^. The fact that populations of cacao have been declining historically is consistent with recent studies that have analyzed the conservation status of more than 15,000 species of Amazonian trees, predicting that *T. cacao* could suffer an additional 50% population decline in the near future^32^. Because of the lack of reliability of these methods to resolve more recent demographics, there is little that can be said about the apparent recent increase in population size. Additional analyses with a larger sample size per population and inference of recent effective population sizes with Identical By Descent (IBD)/linkage-based methods will be necessary to address this issue.

Using what we learned from the admixture/principal components analysis (PCA) (Fig. 2a, b) and our overall assessment of the demographic history of the populations (Fig. 3c, d), we explored the evolutionary history of domestication of the Criollo group from a Curaray ancestor to answer two critical questions: (1) how long ago the ancestral Curaray populations gave rise to what is today known as the Criollo group and (2) the size of the founding population of Curaray ancestry that used to domesticate cacao Criollo in Central America. For this, we analyzed the frequency spectrum of variants under a model of isolation, with migration under a maximum likelihood framework with δaδi^33^. Our analyses show that the fraction of the ancestral Curaray effective population used to domesticate Criollo in Mesoamerica was indeed very small and comprised ~738/1476 individuals (95% CI: 437/574–2647/3894 individuals for mutation rates 7.1 × 10^-9^/3.1 × 10^-9^, respectively; see Supplementary Figures 6 and 7). More importantly, we provide strong support from genomic data analysis that this process started 3600/7200 years before present (95% CI: 2481/4162–10,903/13,806 years before present for mutation rates 7.1 × 10^-9^/3.1 × 10^-9^ mutations bp^-1^ gen^-1^, respectively). The observed distribution of shared variants for different minor allele frequency categories (Fig. 3f) fits well the predicted values under the best fitted model (Fig. 3g) with a distribution of residuals that show a good absolute fit (for details about the alternative demographic models tested see Supplementary Information). Our estimates for the time of separation of the Curaray and Criollo groups overlaps well with archeological evidence and what has been thought to be the onset of cultivation of Criollo in Mesoamerica^8,9,12,34^. These results are consistent with findings of theobromine in Olmec pottery from the capital San Lorenzo as old as the Early Preclassic (1800–1600 BCE)^9,35^. Our demographic analyses are also consistent with large-scale analyses of modern and ancient DNA, which pinpoint the colonization of the American continent by humans to roughly 13,000 years ago^36–38^. In addition, recent analysis of the post-colonization demography of human in South America is consistent with human populations staying in relative low numbers for the first 8000 years and then with the advent of agriculture and thus sedentism, experiencing a population expansion at ~5000 years ago, similar to that experienced during the Neolithic revolution elsewhere on the globe^39^. In short, our understanding of human demographic history suggests that our inference of *T. cacao* domestication in Mesoamerica between 2481 and 13,806 years before present are strongly consistent with the history of human settling in the region, but our knowledge of human history suggests that times closer to the lower limit of the confidence interval or at least lower than 8000 years ago are more likely. A schematic of the best demographic model explaining the data is provided in Fig. 3h. Although we have been as rigorous as possible in the analysis (see Supplementary Figure 8), it will be important to validate the estimated age for divergence between Curaray and Criollo with methods that could better resolve recent demographics with a larger number of individuals from each population.

Our analyses also show that the patterns of linkage disequilibrium (LD) are consistent with the observed demographics, with Criollo populations showing a higher LD over longer stretches of the genome (Supplementary Figure 9 and Supplementary Information)

**Fig. 4:**
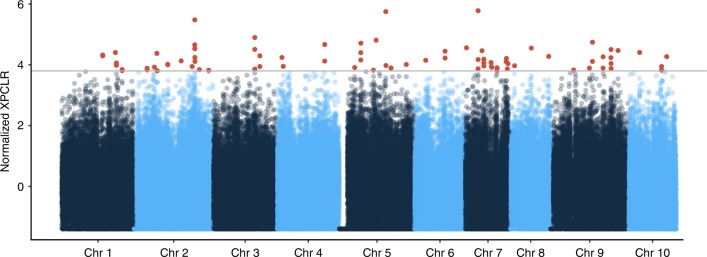
Evidence of positive selection in domesticated *T. cacao*. Maximum likelihood approach for detecting regions of the genome that diverged significantly from the demographic depicted by the site frequency spectrum in Fig. 2e. Red points correspond to windows putatively under selection

One of the greatly appreciated features of the domesticated Criollo cacao is the white cotyledon of the bean, which seems to be associated with desirable flavor qualities. Early work has suggested that decreased concentrations of polyphenols, methylxanthines, and anthocyanin precursors in the cotyledon are associated with this observation^40–42^. Polyphenols and methylxanthines are responsible for the astringency and bitterness detected in cacao beans^43^, and it is thought that the modification of these compounds during the process of fermentation contributes to the final flavor of a chocolate^43^. In fact, during the process of fermentation, the concentration of polyphenols is reduced by up to 70%^44^. Plants of the Criollo variety were likely selected during domestication to reduce this bitterness. We investigated the impact of artificial selection during domestication on the Criollo genome by looking for regions of increased differentiation between Criollo and its sister population Curaray, using XP-CLR, a method that seeks to identify changes in the distribution of allelic variation or changes in the 2D-site frequency spectrum along the chromosomes in sliding windows^45^. We found several regions of the genome in which natural selection has produced higher differentiation between Curaray and Criollo than expected by demographics alone (Fig. 4, Supplementary Data 1 and 2). The most interesting result derives from the identification of genes encoding laccase 14, laccase/diphenol oxidase. Laccases are normally associated with the process of lignification, but it has recently shown that laccases are also involved in the metabolism of polyphenols^46–48^; we hypothesize that selection on these genes likely results in the reduction of the concentration of polyphenols in cacao. We also identify signatures of selection in a region containing the gene encoding xanthine dehydrogenase 1, likely involved in the metabolism of methylxanthines (like theobromine) and also likely to have been the result of the process of selection for reduced bitterness^42^. An additional list of genes in regions identified to be under selection is provided in Supplementary Table 3 and includes genes involved in genomic stability (structural maintenance of chromosomes), disease resistance, abiotic stress response (WRKY DNA-binding protein), transcriptional regulation (MYB domain), and signaling (cysteine-rich RLK receptor as well as S-domain-2 5 genes).

Most mutations that appear in the genome are deleterious and have the potential for reducing reproductive success^49–51^. The fate of these mutations and their transit time in a population strongly depends on the intensity of genetic drift, purifying selection, and the degree of dominance of the mutations. Mathematical population geneticists were long concerned with the impact of the accumulation of deleterious mutations in a population^52^. The process of domestication in animals and plants has been used as a framework to study how intense selection of some desirable traits affects the accumulation of deleterious mutations in the population^3,53^. Yet, thus far, we have little evidence of how the process of accumulation of deleterious mutation affects traits associated with fitness or, in the case of crops, productivity.

### Test of the cost of domestication hypothesis in *T. cacao* L.

**Fig. 5:**
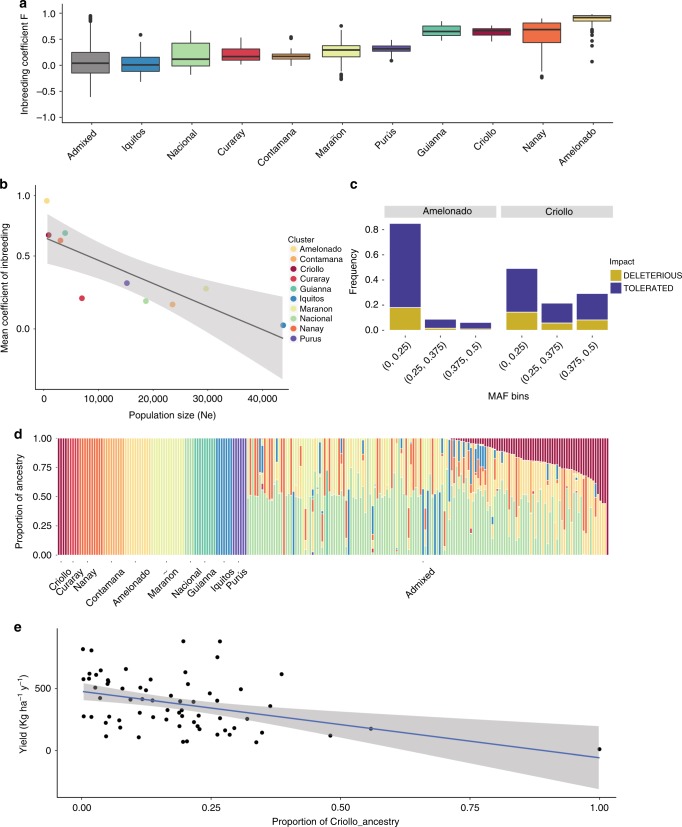
Accumulation of deleterious mutations during domestication in *T. cacao*. **a** Distribution of coefficients of Inbreeding (F) per population (including the group of Admixed individuals). **b** Coefficients of Inbreeding as a function of the harmonic mean of the effective population size (estimated from the median PSMC shown in Fig. 2D, model: F ~ Ne+ Group, *p* < = 0.003, r^2^ = 0.9). **c** Distribution of deleterious/tolerated mutations inferred with SIFT for the Criollo and Amelonado groups for rare and two classes of common binned minor allele frequency classes showing the highest relative proportion of common deleterious and tolerated amino acid changes in Criollo. **d** Population structure inferred using a maximum likelihood under a supervised model for an independent set of genotyped individuals (see supplements) for which productivity has been measured. **e** Productivity (measured as Kg of beans per hectare per year) as a function of Criollo ancestry in the newly genotyped set of individuals; the results show a significant reduction in productivity as the proportion of Criollo ancestry increases, after correcting for inbreeding

Populations of cacao have been declining over time and a natural consequence of the reduction in population size is increasing in inbreeding. Because all ten populations of cacao are experiencing reductions in population size, it is expected that this process will have a similar effect across populations, and the differences in magnitude of inbreeding will reflect differences in population size. We observe an increase in the amount of inbreeding (estimated as F-statistics^54^) when the Admixed cluster of individuals (expected to have low inbreeding) is compared with the ten populations defined in Fig. 1 (Fig. 5a, Kruskal–Wallis test chi-squared = 803.45, df = 10, *p*-value < 2.2e^−16^, significant Nemenyi’s post-hoc tests between Admixed and all populations, except Iquitos and Nacional). These differences between the coefficients of inbreeding can be partially explained as a function of the differences in historical population size among genetic clusters (Fig. 5b, see Supplementary Information).

Population genetics theory predicts that selfing increases the efficiency in the elimination of recessive deleterious mutations, when compared with outcrossing populations, because variants otherwise hidden in heterozygous individuals will be exposed to the action of natural selection^55,56^. In contrast, domestication is a process that has been shown to contribute to the maintenance of deleterious mutations in higher frequency in populations^3,53^. The impact of domestication on arboreal crops is not well understood, and it is even less understood in a plant-like cacao that in domesticated varieties utilizes self-compatibility, a mechanism that tends to purge deleterious mutations. In order to test the impact of selfing and domestication on the accumulation of deleterious mutations in cacao, we annotated amino acid changes in *T. cacao* based on phylogenetic conservation (as implemented in SIFT4, see Methods) either as tolerated or deleterious (see Supplementary Methods).

We inferred the distribution of deleterious/tolerated changes for combined categories of minor allele frequency in Amelonado and Criollo. The inference of a mutation being deleterious or tolerated for each gene model was done with a method that assesses phylogenetic conservatism in polymorphic changes, using SIFT4^57,58^. Amelonado was used to generate an expectation on the accumulation of deleterious mutations for a scenario with strong selfing (similar to Criollo) in the absence of strong domestication to understand the impact of domestication in the distribution of deleterious/tolerated in Criollo (Fig. 5c). Amelonado presents a distribution of deleterious/tolerated mutations with a high frequency of rare variants and a reduced representation of variants in intermediate and large minor allele frequencies. This is consistent with most deleterious mutations being purged by selfing in the population. On the other hand, we observed significant, larger counts of deleterious mutations in higher minor allele frequency classes in Criollo (Fig. 5c and Supplementary Figure 10 and Supplementary Table 5), an observation that was significant across frequency classes (Mantel–Haenszel test, *p-*value < 2.2e^−16^, Supplementary Figure 10). These differences indicate that selfing in the Criollo populations (when combined with a strong genetic drift due to the domestication process in Central America) has not been a strong enough to purge deleterious mutations in the population, as was apparent in Amelonado, despite being a predominant form of mating. Similar patterns of accumulation of deleterious mutations during the process of domestication have been reported in animals and plants, such as in the comparison of teosinte and domesticated maize^59^, but is reported here for the first time for an arboreal crop. In most of the analyses that have been performed to date in other organisms, including dogs and humans^53,60^, it has not been shown what the impact of the accumulation of deleterious mutations is on fitness. We tested the hypothesis that the accumulation of deleterious mutations due to domestication would decrease fitness by examining the relationship between Criollo ancestry and a measure of performance in cacao using an independent dataset. Individuals were genotyped with a SNP array that was developed using a subset of genotypes inferred as part of this work and published elsewhere^61^. We measured bean (seed) productivity (yield in kilograms of bean per hectare per year for each plant) as a measure of fitness. We inferred proportional ancestry to a new set of admixed individuals for which productivity had been assessed (Fig. 5d and Supplementary Figure 11) and showed that there is a significant negative relationship between Criollo ancestry and fitness (Fig. 5e, with Criollo ancestry decreasing yield per hectare per year in ~319.9 units per percent unit of ancestry, *p* = 0.000425, additional details for the model are available in the Supplementary Information). We also demonstrate that despite the decrease in fitness in domesticated populations, there is no loss in quality and ability to prepare chocolate from its beans (Supplementary Figure 12).

In summary, we provide the first general view of how natural diversification has shaped genetic variation in *Theobroma cacao*. We provide the first comprehensive view of the demographic scenario involved in the domestication of the Criollo variety and identify genes that could function in the desirable taste attributes of the Criollo variety. More importantly, we show how genomic resources can be successfully used to evaluate how the process of domestication has shaped the pattern of accumulation of deleterious mutations in arboreal crops, impacting fitness in a considerable way, validating for this species the cost-of-domestication hypothesis^20^.

## Conclusions

Our study has provided a closer look at the evolutionary history of *Theobroma cacao* L. We have developed a great resource for breeders and researchers, which include over 7 M SNPs, and corresponding genomic annotation for those variants. The results of the work presented in this manuscript shed light on a diverse array of questions that range from a deeper characterization of the genetic population structure in cacao populations to increasing our understanding of the evolutionary history of domestication in cacao. Most importantly, our work has provided strong genomic evidence supporting the cost-of-domestication hypothesis, stating that the process of improvement and selection for desirable traits is hindered by the undesired accelerated accumulation of deleterious mutations.

## Methods

### Sampling

We sampled leaves from accessions at the Cacao Research Unit at the University of West Indies and CATIE in Costa Rica (Supplementary Table 1).

### DNA extraction and sequencing libraries preparation

Samples processed at Stanford University were prepared as follows:

DNA was extracted using ZR Plant/Seed DNA MiniPrep™ (Zymo Research Inc). Approximately 3 g of leaf material per extraction per sample was cut and placed in homogenization tubes with ceramic pearls and lysis buffer. Samples were homogenized in a FastPrep-24^TM^ (MP Biomedicals, LLC) placed in a cold room at 4 °C for 60 s at a speed of 4.5 m sec^-1^. If the tissue was not homogenized thoroughly, the tissues were homogenized for an additional 20–40 s at the same speed. DNA was quantified using a Qubit^TM^ 3.0 fluorometer (ThermoFisher Scientific), using a dsDNA HS Assay Kit. Additionally, overall quality of extracted DNA was assessed with 2% E-Gel (Invitrogen, Carlsbad, CA). Most of the samples were prepared using Nextera DNA Sample Preparation Kits (Epicentre, Chicago, IL, USA) and NEBnext® Ultra DNA Library Prep Kit for Illumina (New England BioLabs, Inc). The remaining samples were prepared by first shearing the genomic DNA using a M220 Focused-ultrasonicator™ (Covaris Inc) and NEBnext® Ultra DNA Library Prep Kit for Illumina (New England BioLabs, Inc). Libraries were quantified on Agilent 2100 Bioanalyzer High Sensitivity DNA chip for concentration and size distribution, pooled in sets of 3–4 per batch, and sequenced on the HiSeq 2000/2500 platform at the Stanford Sequencing Service Center (100 cycles, paired read mode).

Samples processed at Indiana University were prepared as follows:

DNA was extracted using a protocol customized for enrichment of high molecular weight DNA from cacao leaves. Approximately 450 mg of leaf material per sample was ground to powder under liquid N_2_ using mortar and pestle. Tissue powder was homogenized and washed twice by vortexing in 3 ml of ice cold 100 mM HEPES, 0.1% PVP-40, 4% b-mercaptoethanol, followed by centrifugation at 7000 rpm in an Eppendorf F35-6-30 rotor. Nuclei were extracted from tissue pellets on ice in 50 mM Tris-Cl pH 8.0, 50 mM EDTA, and 50 mM NaCl with 15% sucrose, and centrifuged at 3600 rpm to pellet trace the cellular debris. Nuclei were lysed at 70 °C for 15 min in 20 mM Tris-Cl pH 8.0, 10 mM EDTA with the addition of SDS to a final concentration of 1.5%. Protein was precipitated on ice with the addition of NH_4_OAc to a final concentration of 2.7 M, pelleted twice by centrifugation at 7000 rpm. DNA was precipitated using gentle inversion in an equal volume of cold isopropanol, followed by centrifugation at 7000 rpm. DNA pellets were washed in 70% ethanol and resuspended in 10 mM Tris-Cl, 1 mM EDTA using wide bore pipette tips. DNA quality and quantity in the high molecular weight fraction (24 to ≥ 60 kb) was assessed by migration on Genomic DNA Screen Tape, Agilent TapeStation 2200 Software (A.01.04) (Agilent) and secondarily quantified by fluorimetry using the dsDNA HS Assay Kit (Invitrogen) with a Qubit^TM^ 2.0 fluorometer (ThermoFisher). Sequencing libraries were prepared either as unamplified NGS libraries, using the PCR-free DNA library kit (KAPPA) or minimally amplified libraries were prepared using the TruSeq DNA Sample Prep Kit (Illumina) with four cycles of PCR at the Roy J. Carver Biotechnology Center, University of Illinois at Urbana–Champaign (UIUC). All library preparation steps were according to the manufacturer with the exception that after shearing for minimally amplified libraries, DNA was cleaned through a Zymo column and size selected to retain only 400–600 bp fragments. All libraries were evaluated for quality using an Agilent 2100 Bioanalyzer High Sensitivity DNA Assay (Agilent), quantified by qPCR, pooled in sets of 12 at equimolar concentration, and sequenced as paired 2 × 161nt reads on a UIUC HiSeq2500 instrument using HiSeq SBS sequencing kit version 4. Fastq files were generated with CASAVA 1.8.2.

### Read processing and SNP identification

The Illumina data were basecalled using Illumina software CASAVA 1.8.2, and sequences were demultiplexed with a requirement of full match of the six nucleotide index that was used for library preparation. Samples prepared using Nextera were hard clipped 13 nt from the 5’ end. Following demultiplexing, raw sequenced data was analyzed for quality using FastQC^62^. We performed adaptive quality trimming (setting a quality threshold of 25) and additional hard trimming of the reads based on stabilization of the base composition on the 5’ end of the sequences using TrimGalore! and cutadapt^63,64^. Sets of reads from individual samples were mapped to the Matina-v1.1 reference genome^21^, using the burrow-wheeler aligner BWA^65^ with relaxed conditions for the editing distance (0.06), as it was expected that *T. cacao* has a high genetic diversity. Aligned sam files were preprocessed prior to performing SNP identification with Samtools/Picard Tools and Bamtools^66–68^ to mark duplicates, fix mate pair information, correct unmapped reads flags, and obtain overall mapping statistics. We followed recommendations of the Genome Analysis Toolkit to perform base quality recalibration and local realignment to minimize false positives during the SNP calling procedure^69^. Finally, we performed genotype calling using Real Time Genomics population analysis tool to speed the process of SNP identification^70^. Calls were also called with GATK, and a suitable subset of SNPs were kept after a combination of Variant Quality Score Recalibration (VQSR) and hard filters that included thresholds in coverage (maximum coverage = 200*50×), quality by depth (QD 2) estimated from the division of variant confidence by unfiltered depth of non-reference samples, fisher strand test (FS 50), and the root mean square of the mapping quality across samples (MQ 30). Variants identified were phased, per population, using shapeit v2.12 on a subset of variants in which the minor allele frequency (MAF) > 0.05^71,72^. The phasing was performed per chromosome for the ten main chromosomes using only biallelic sites.

Identified SNPs were annotated using SNPEff^73^. For this, we used the current gene annotation from the Matina-v1.1 reference genome^21^ to construct a new database for *Theobroma cacao*. This database was used to annotate the observed polymorphisms following their potential effect on gene expression and functionality according to their position with respect to the coding regions.

### Population genetic analyses

We characterize the distribution of genetic variation in the populations, estimating variation using two approximations for the inference of genetic variation: Watterson’s theta (θ_w_)^74^ and the number of pairwise differences per site (π)^75^. We used vcftools^76^ to estimate both statistics in windows of 1 kb. Generalized Linear models to explain the differences in diversity among populations are explained in the section *Distribution of Genetic variation among genetic groups* in the Supplementary text.

We used a ADMIXTURE^24^, an implementation of an approach similar to well-known STRUCTURE^77^. Based on an expectation–maximization algorithm, ADMIXTURE uses a maximum likelihood-based approach to assign ancestry genome wide and visualize the genetic structure of the *T. cacao* populations. A cross-validation procedure is employed to select the most likely number of clusters that explains the structure of the data^24^. We filtered our data and restricted our analysis to SNPs with minor allele frequency over 5%, and we also pruned the data for LD as the approximations assume unlinked loci. For this, we used vcftools^76^ to estimate LD (r2) scores for each pair of SNPs in windows of 2000 SNPs and excluded one of the pair if r^2^ > 0.45. The windows were selected with 500 SNPs of overlap. The final dataset contained 63,374 SNPs. We analyzed this dataset using *ADMIXTURE* and set 2–18 ancestral populations (*K* = 2 to *K* = 18) in 100 replicates. We checked for convergence of individual *ADMIXTURE* runs at each *K* by evaluating the maximum difference in log likelihood scores in fractions of runs with the highest log likelihood scores at each *K*. We assume that a global log likelihood maximum was reached at a given *K* if at least 10% of the runs with the highest score show minimal variation in log likelihood scores and present consistent assignment to the groups. It has been shown^78^ that a threshold of 5 log likelihood units is conservative enough to assure similar results to those obtained with *CLUMPP*^79^. In addition to the admixture analysis, we performed a multidimensional scaling analysis on the same set of SNPs employed for *ADMIXTURE*. First, we normalized the data (centered and standardize) following previous recommendations^80^ and performed MDS analyses using Singular Value Decomposition on the normalized data using the cmd scale function in R.

We measured population differentiation resulting from restrictions in gene flow between populations using Weir and Cockerham’s F_ST_ estimator^81^ in windows of 5 kb, after filtering out low-frequency alleles. To summarize the genome-wide differentiation among populations, we estimated the mean of F_ST_ estimators across windows and standard error for every pair of comparisons.

The map and location of populations in South America was created using ggmaps in R. The maps used in ggmaps are obtained from Google maps (open access source) and the diamonds used for the positioning of the populations were modified to increase the size in the Illustrator.

We fitted a generalized linear model to explain the differences in genetic diversity along the Pacific/Atlantic axis of genetic differentiation captured in the second component of a multidimensional scaling. For this, we estimated the centroids for PC1 and PC2 of the data presented in Fig. 1b. These centroids were used as predictors (*β*_*i*_) to explain the differences in mean genetic diversity per population (measured as π, *Y* in the following model) under a simple linear model with a Gaussian family $Y = \beta _o + \beta _i + \epsilon$. Admixed individuals were excluded from the analysis.

We used a model-based approach to infer the population relationships between the ten main groups as implemented in TreeMix^26^ to identify the relationships between populations and identify signatures of domestication.

We used two methods to infer the demographic history of populations using individual genomes and small sets of individuals per population. First, we used the pairwise sequentially Markovian coalescent as implemented in PSMC;^28^ second, we used SMC++, a likelihood-free method that can leverage information from multiple individuals from the population (as opposed to PSMC) to infer population size changes in the past^29^. We assumed a mutation rate μ = 7.1 × 10^−9^ *mutations* *×* *bp*^*-1*^ *×* *gen*^*-1*^^82. 83^. We also examined the effect of uncertainty in mutation rates by including analysis following recent work suggesting that mutation rates could be half of that estimated previously on the order of 3.1 × 10^−9^ *mutations* *×* *bp*^*−1*^  *×* *gen*^*−1*^^84^. Additional details are provided in the Supplementary text. We assumed a generation time of 5 years, based on the observation that it takes 5 years on average to go from seed to seed in cacao. The figures describing the evolutionary history inferred with PSMC were obtained from adjusting a smoothing spline across individual histories inferred for each sample that corresponded to the same population.

We estimated inbreeding using a simple moment estimator F = 1 – Het_obs_/He_exp_^85^ to assess the magnitude of inbreeding experienced by individuals in each population. We then addressed the impact of historical population size on estimated inbreeding using an ANOVA to compare the estimated inbreeding F-statistics among populations.

The association between effective population size and inbreeding was examined with a generalized linear model of the form $Y = \beta _0 + \beta _i + {\it{\epsilon }}$, where Y is the inbreeding coefficient F, β_0_ is the intersect, and β_i_ is the effect of effective population size. As a predictor, we used the harmonic mean of the effective population sizes estimated under the PSMC model for each population under the population genetic assumption that the smallest population size experienced by the population will strongly influence the magnitude of drift.

Using the inferred relationships among populations obtained with TreeMix, we selected the most closely related population to domesticated Criollo (the Curaray population) to perform detail demographic analyses and infer time of divergence between populations and demographic trajectories for the populations. We use an approximation based on the comparison of the observed site frequency spectrum and simulations in a maximum likelihood framework to decide which model better explained the data, as implemented in the program δaδi^33^. We informed the three main models tested (see Supplementary Information) with the aid of the PSMC results. Akaike information criteria and magnitude of the residuals were employed for model selection. For the estimation of confidence intervals, we performed 1000 bootstraps of the observed dataset and performed estimations using the selected demographic model. Additional details on the estimation of confidence intervals, uncertainty of the generation time and mutation rates, and detailed analysis of the likelihood surface for parameters of interest are provided in the Supplementary Information.

Regions under selection were inferred by analyzing departures from the site frequency spectrum. Analyses performed with XP-CLR^45^ allowed us to detect local deviations from the genome-wide site frequency spectrum. For this, we set fixed windows of 0.05 cM for 200 SNPs and grid size of 2 kb. For these analyses, we used the Curaray population as reference and took the top 1% windows with significant XP-CLR score. In addition, we selected those windows of 5 kb in which F_ST_ values corresponded to the top 1% of the distribution to examine regions of the genome which potentially present higher differentiation than expected.

### Cost-of-domestication analysis

We inferred deleterious and tolerated effects for non-synonymous mutations using a method that uses phylogenetic conservatism. To deploy this method as implemented in Sorting Intolerant from Tolerant (SIFT) 4G^58^, we built a custom database of predictions for all possible non-synonymous SNPs using SIFT4G for *T. cacao*. SIFT outputs a SIFT score for each amino acid substitution; the score ranges from 0 to 1. The amino acid substitution is predicted deleterious if the score is ≤ 0.05 and tolerated if the score is > 0.05.

We used a log-linear model to test for differences in the number of deleterious and tolerated mutations between Criollo and Amelonado. Amelonado was chosen because of the similar levels of inbreeding observed. Because of the differences in sample size, we estimated for each population the number of deleterious and tolerated mutations at three different allele frequency classes: rare (0–0.25), intermediate (0.25–0.375), and frequent (0.375–0.5). This model allowed us to test for general trends in the data and show that there is a significant difference in the number of deleterious mutations among Criollo and Amelonado along binned classes of minor allele frequency. A post-hoc analysis was done with the Mantel–Haenszel test to test for specific effects. See Supplementary information for additional details on the implementation.

Finally, we genotyped an additional set of 151 accessions using a customized chip of 15 K SNPs specific for cacao that was developed in parallel to this work using the novel variants identified in a subset of the accessions^61^. We intersected the genotyped set with the 79 accessions from this work that clearly belong to each one of the putative genetically differentiated populations and performed a supervised ancestry analysis in ADMIXTURE with conditions similar to those explained previously. We measured productivity (measured in *kg* *×* *ha*^*−1*^ *×* *yr*^*−1*^) in all 151 accessions. The impact of the accumulation of deleterious mutations on productivity was assessed by fitting a generalized linear model to explain productivity (measured in kg × ha^−1^ × yr^−1^) as a function of Criollo ancestry after correcting for inbreeding (Supplementary Information for more details). We built a generalized linear model with a Gaussian family of the form:

*Y*= *β*_0_+*β*_1_+*β*_2_+*ε*, where *Y* corresponds to the yield, *β*_0_ corresponds to the intersect, *β*_1_ corresponds to the proportion of Criollo ancestry, and *β*_2_ is the coefficient of inbreeding F estimated for each individual.

We compared the estimates obtained when Criollo ancestry is used versus those obtained when Amelonado ancestry is used as a predictor to test for the specific effect of domestication and not just inbreeding. Supplementary Figure 13 shows the results of association analysis between Amelonado ancestry and productivity. Additional details about the analysis are provided in the Supplementary Information.

### Code availability

The computer code is available by OEC via a github repository oeco28/Cacao_Genomics at https://github.com/oeco28/Cacao_Genomics/

## Electronic supplementary material

Supplementary Information

Description of additional supplementary items

Supplementary Data 1

Supplementary Data 2

## Data Availability

SNPs are available at the European Variation Archive under accession codes PRJEB28591 (project) and ERZ696780 (analyses). The raw sequencing data is accessible at the SRA from ncbi via the BioProject PRJNA486011.
