# Supplementary material for: Population genomic analyses of the chocolate tree, Theobroma cacao L., provide insights into its domestication process
Source: Commun Biol. 2018 Oct 16;1:167. doi: 10.1038/s42003-018-0168-6 (PMC6191438; doi:10.1038/s42003-018-0168-6)
Supplement: Supplementary file 1 — Supplementary Information [file 42003_2018_168_MOESM1_ESM.pdf]

Supplementary Information for

Genomic insights into the domestication of the chocolate tree, *Theobroma cacao* L.

Omar E. Cornejo[#], Muh-Ching Yee, Victor Dominguez, Mary Andrews, Alexandra Adams, Erika Strandberg, Donald Livingstone III, Conrad Stack, Pathmanathan Umaharan, Alberto Romero, Stefan Royaert, Nilesh R. Tawari, Pauline Ng, Osman Gutierrez, Wilbert Phillips, Keithanne Mockaitis, Carlos D. Bustamante[#], Juan C. Motamayor[#].

*Correspondence to: juan.motamayor@effem.com, cdbustam@stanford.edu

Table of contents

Supplementary Figures …………………………………………………………… ………2

Supplementary Tables ……………………………………………………………………….13

Supplementary Methods ………………………………………………… ……………...…67

Supplementary Results and Discussion ……………………………………………………..71

Supplementary References. ………………………………………………………………81

Supplementary Figures

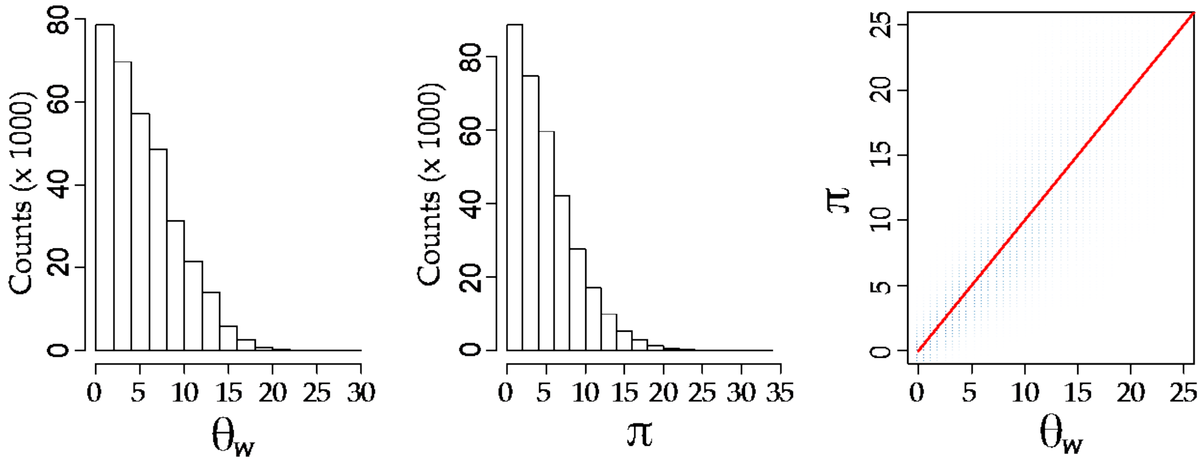

Supplementary Figure 1 | Left and center panels present the distribution of Watteron's θ_w per Kb, and π per KB respectively. Right hand panel presents the scatterplot of Watterson's θ_w, and π; ther red line corresponds to the 1:1 relationship between the two.

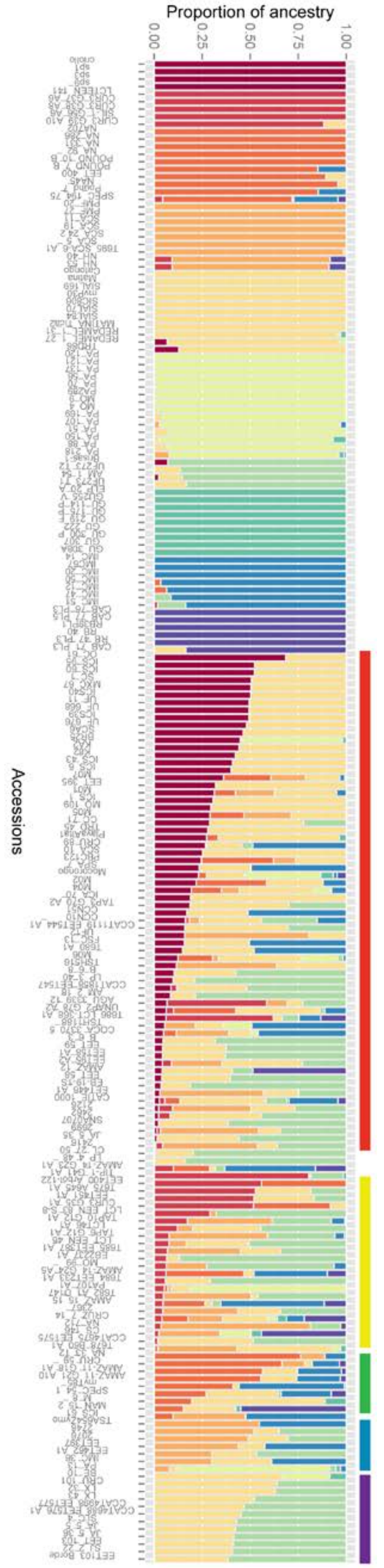
Proportion of ancestry
0.00
0.25
0.50
0.75
1.00
Accessions

Supplementary Figure 2 | Ancestry assignment (K=10) for all admixed individuals. The order of the groups corresponds to the assignment in Figure 1 of the main manuscript. From left to right, the colors correspond to the following groups: Criollo (dark red), Curaray (red), Nanay (dark orange), Contamana (orange), Amelonado (light orange), Marañon (light green), Nacional (green), Guianna (dark green), Iquitos (blue) and Purus (purple). The color bars on top of the admixed individuals correspond to each of the arbitrary groups defined for cacao on this work (see supplementary text) to help the work of breeders.

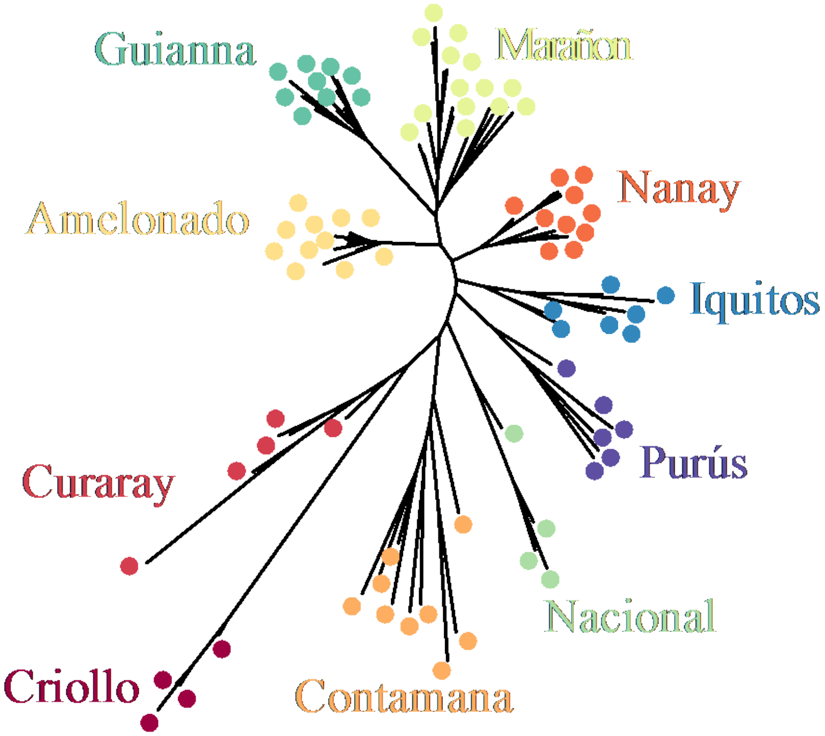

Supplementary Figure 3 | Neighbor-Joining phylogenetic tree among accessions belonging to each one of the 10 main populations described for cacao.

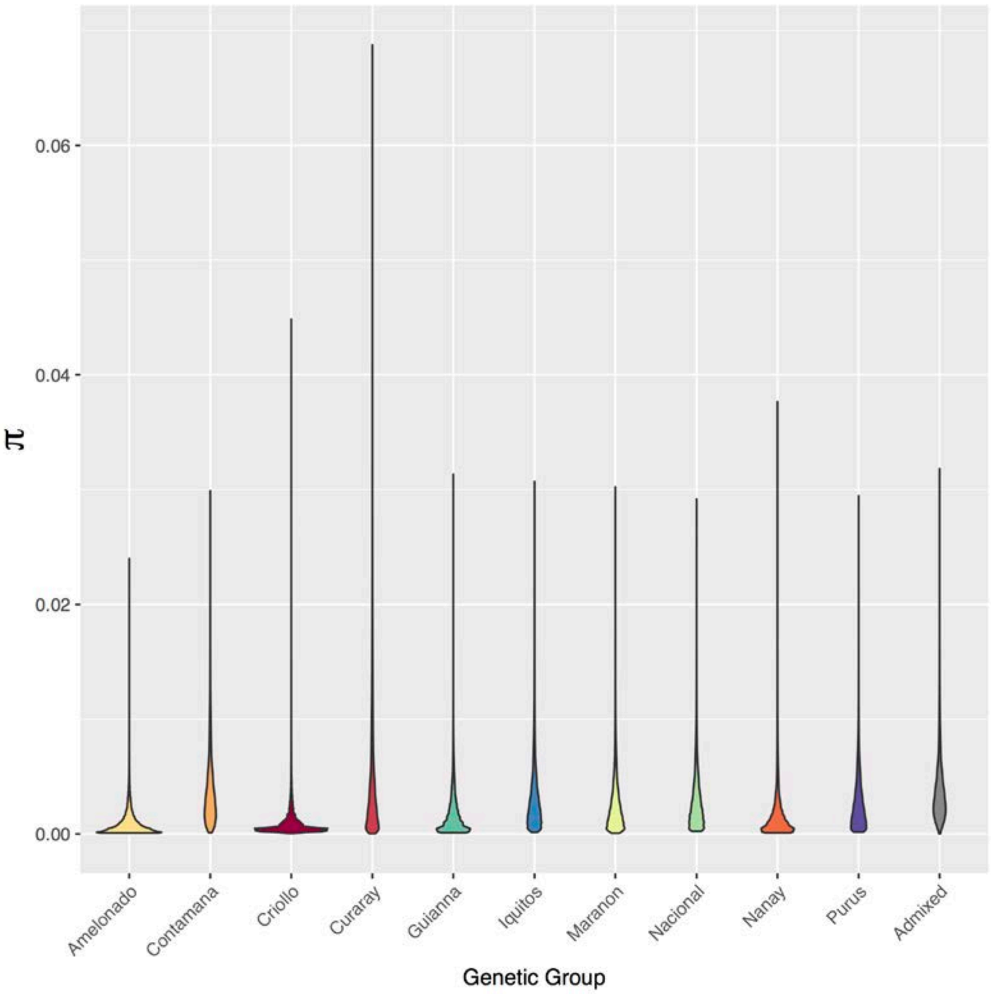

Supplementary Figure 4 | Distribution of genetic diversity (measured as π), represented as violin plots. Differences in overall genetic diversity among groups is significant (see model fitting in text).

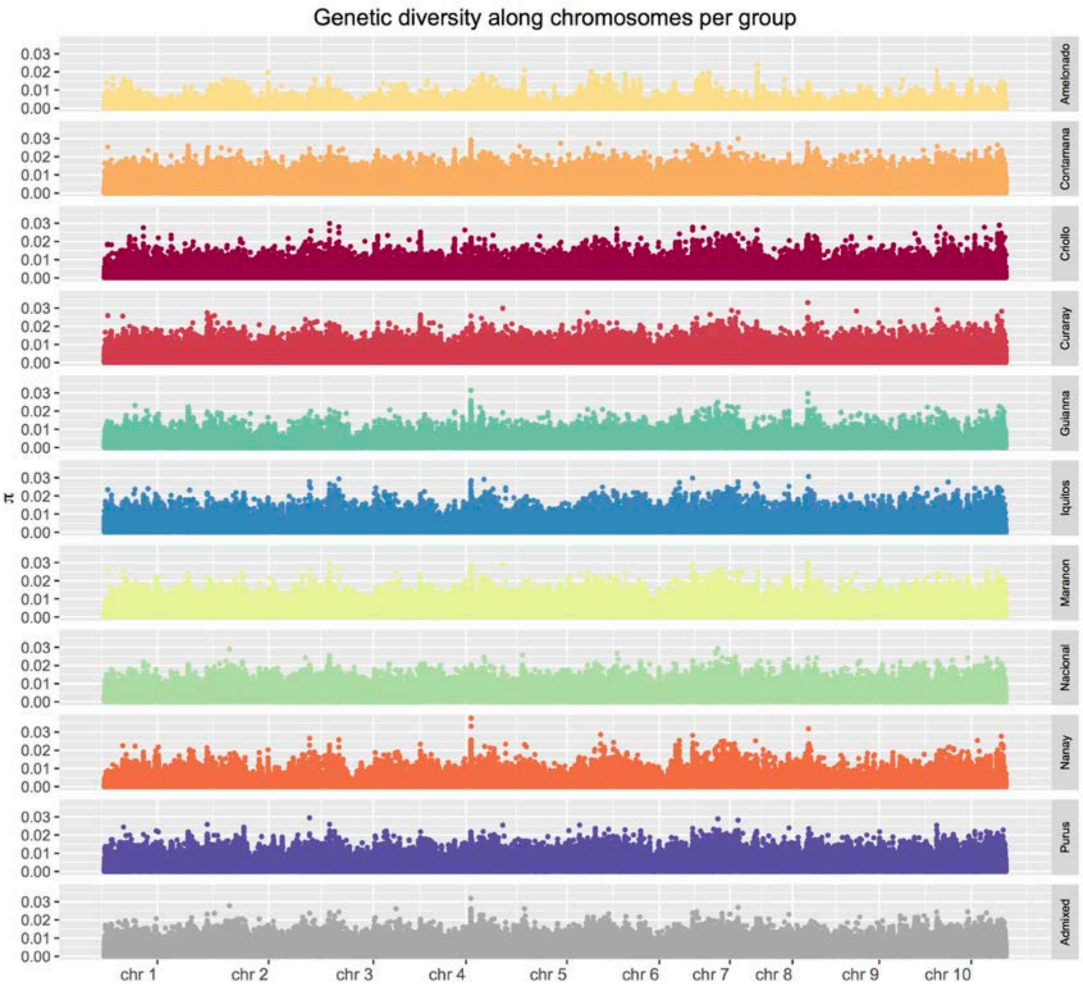

Supplementary Figure 5 | Distribution of genetic diversity (measured as π per base) along the genome for each genetic group (including admixed individuals).

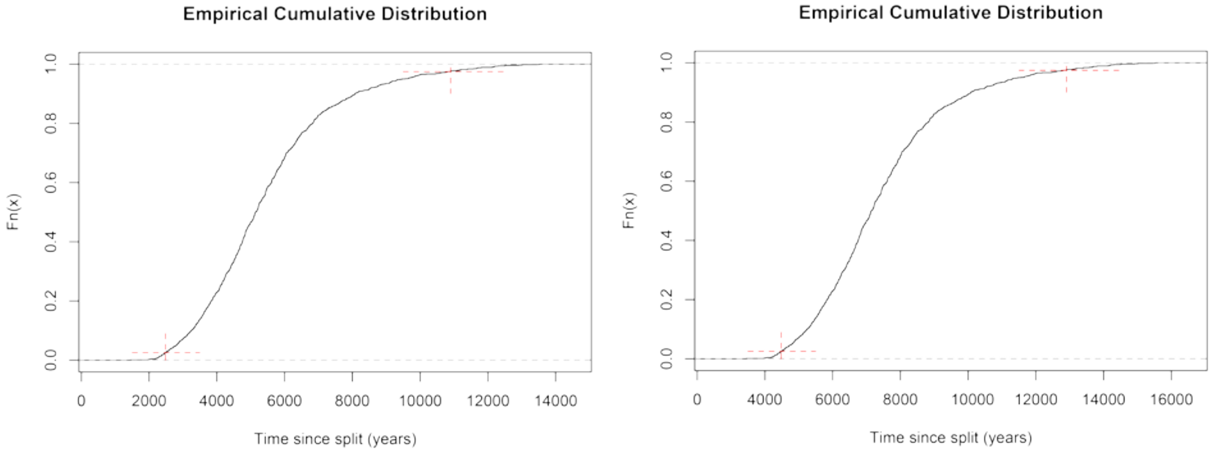

Supplementary Figure 6 | Empirical cumulative distribution for the time since split of Curary and Criollo populations. Red crosses indicate the 95% confidence boundaries. Left panel for mutation rates of 7.1×10^{-9} changes per site per generation. Right panel for mutation rates of 3.1×10^{-9} per site per generation.

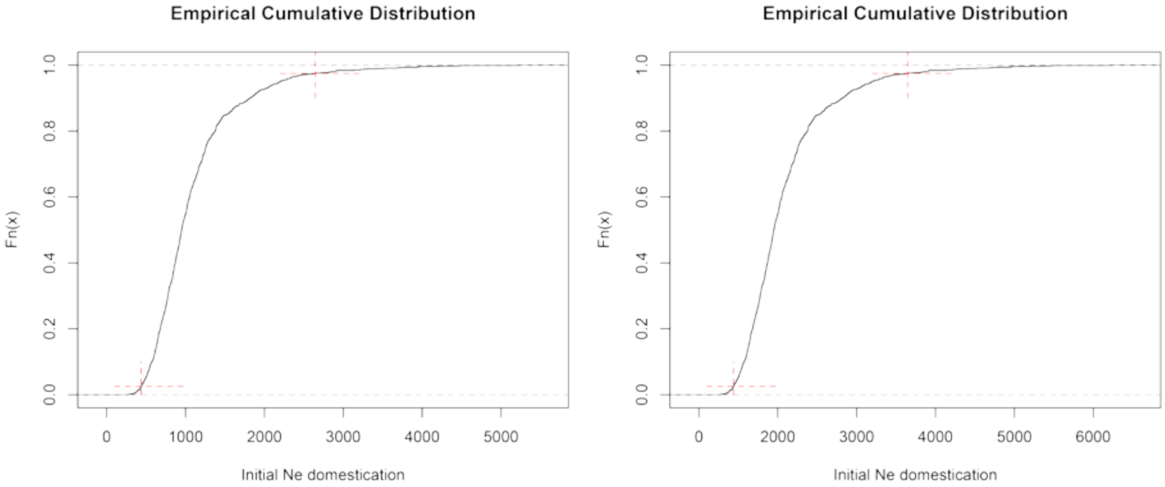

Supplementary Figure 7 | Empirical cumulative distribution for the fraction of ancestral Curary population used for domestication of the Criollo variety. Red crosses indicate the 95% confidence boundaries. Left panel for mutation rates of 7.1×10^{-9} changes per site per generation. Right panel for mutation rates of 3.1×10^{-9} per site per generation.

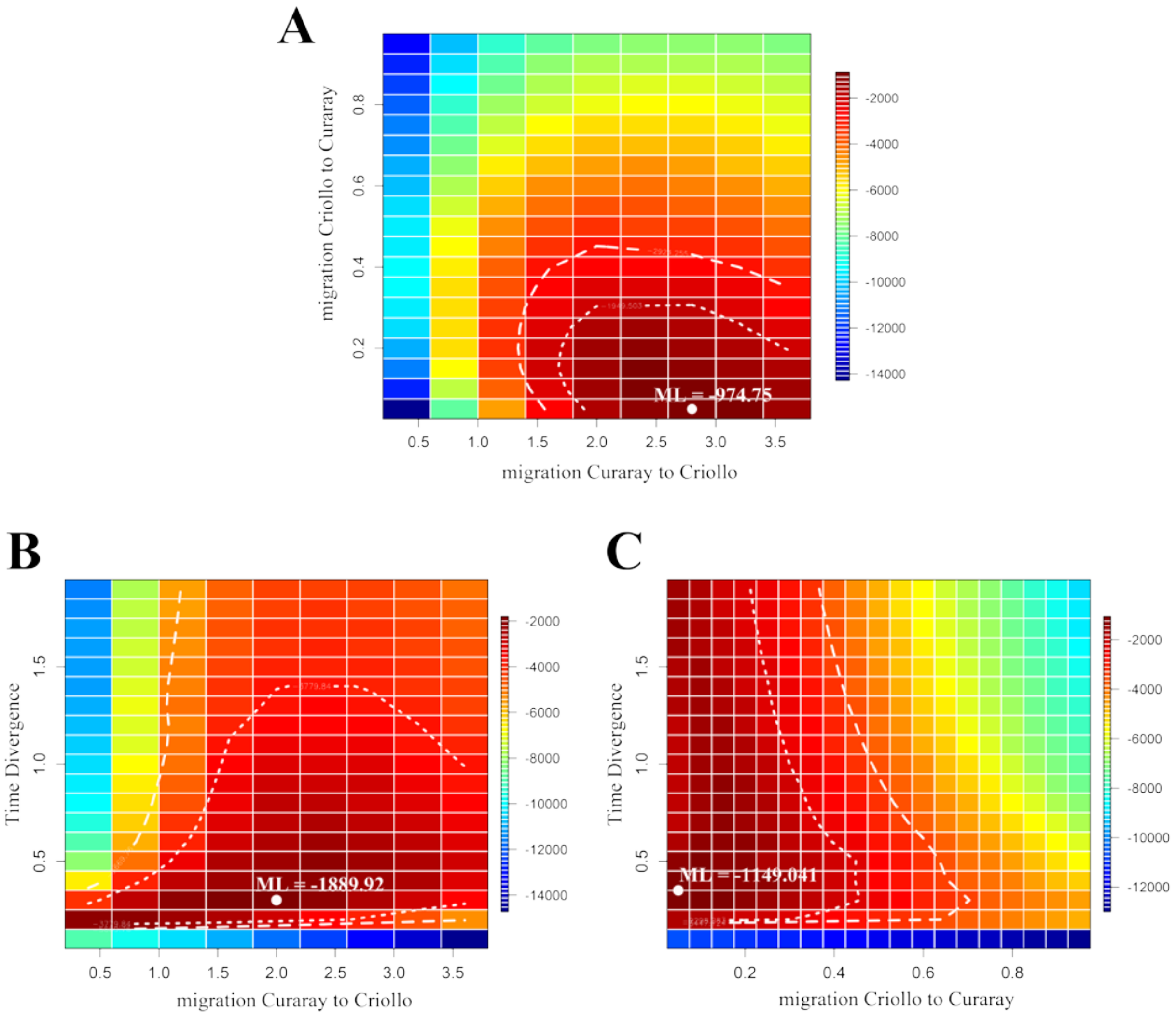

Supplementary Figure 8 | Likelihood profiles for different combinations of parameter values: A) migration from Curaray to Criollo (x-axis) and Criollo to Curaray (y-axis); B) migration from Curaray to Criollo (x-axis) and divergence time between Criollo and Curaray (y-axis); and C) migration from Criollo to Curaray (x-axis) and divergence time between Criollo and Curaray (y-axis). In all cases, maximum likelihood (ML) values are shown with a white dot and the value in text. The dotted line correspond to the area for combinations of parameters 2 likelihood units smaller than ML and the dashed line 3 likelihood units smaller.

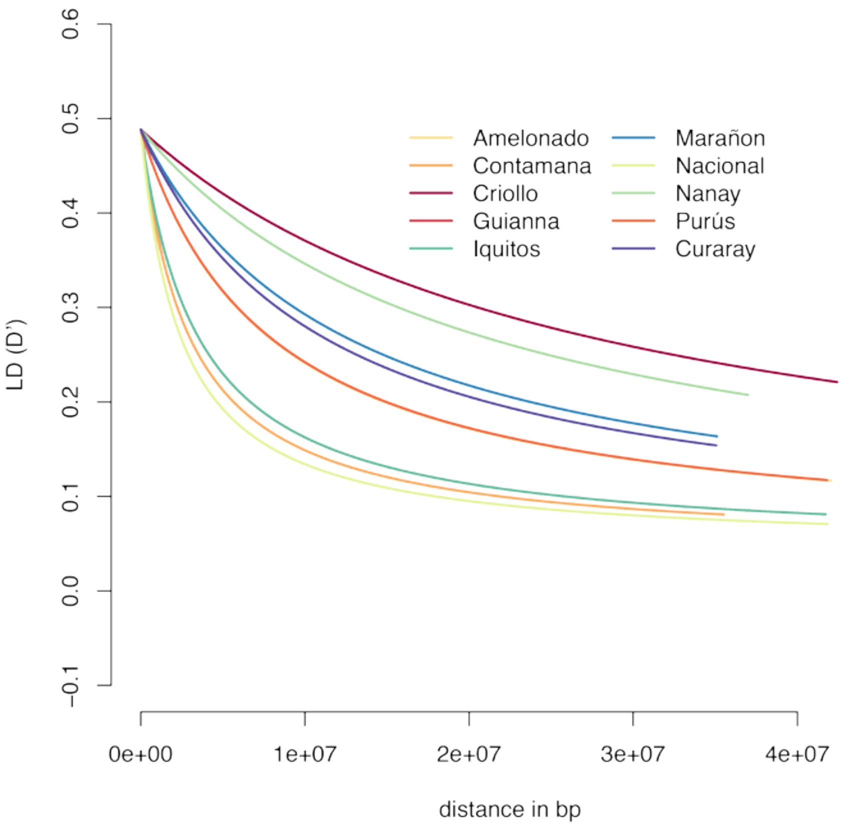

Supplementary Figure 9 | LD decay plots for each genetic group.

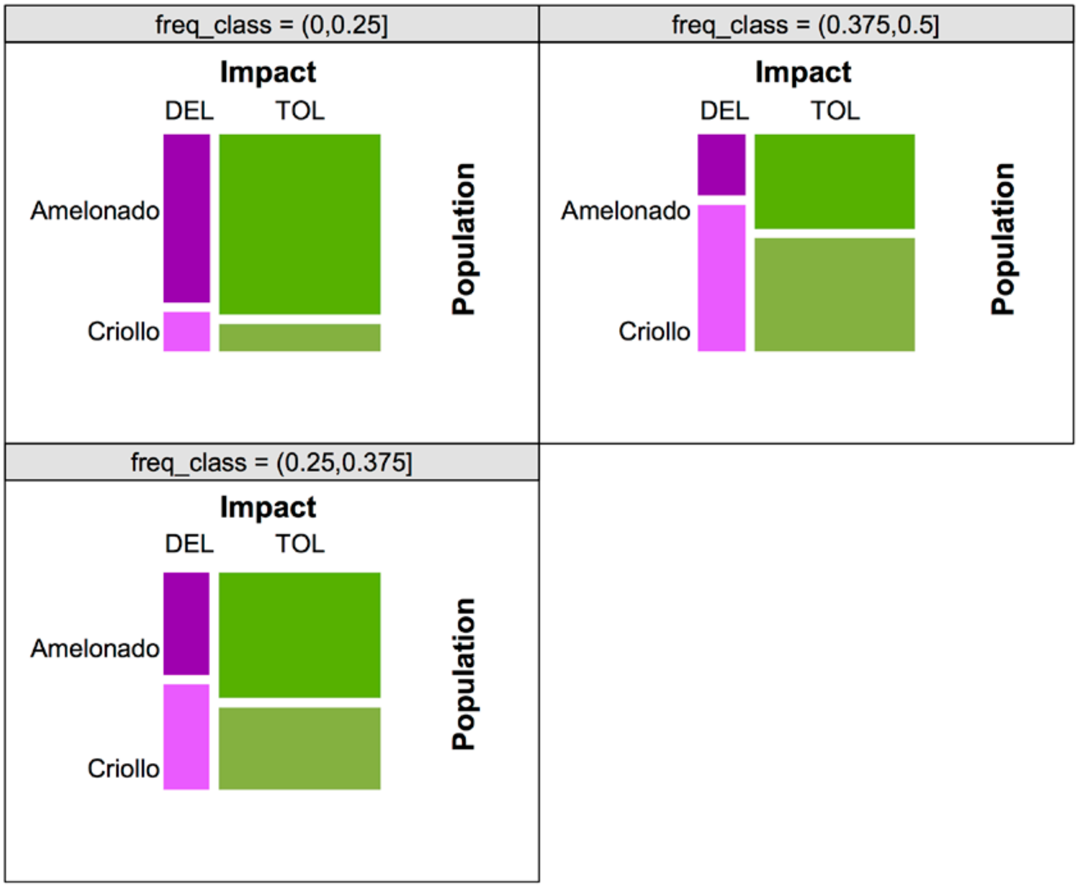

Supplementary Figure 10 | Mosaic plot showing the proportional distribution of deleterious (DEL; shades of magenta) and tolerated (TOL; shades of green) among Criollo (light colors) and Amelonado (darker colors) for each minor allele frequency class.

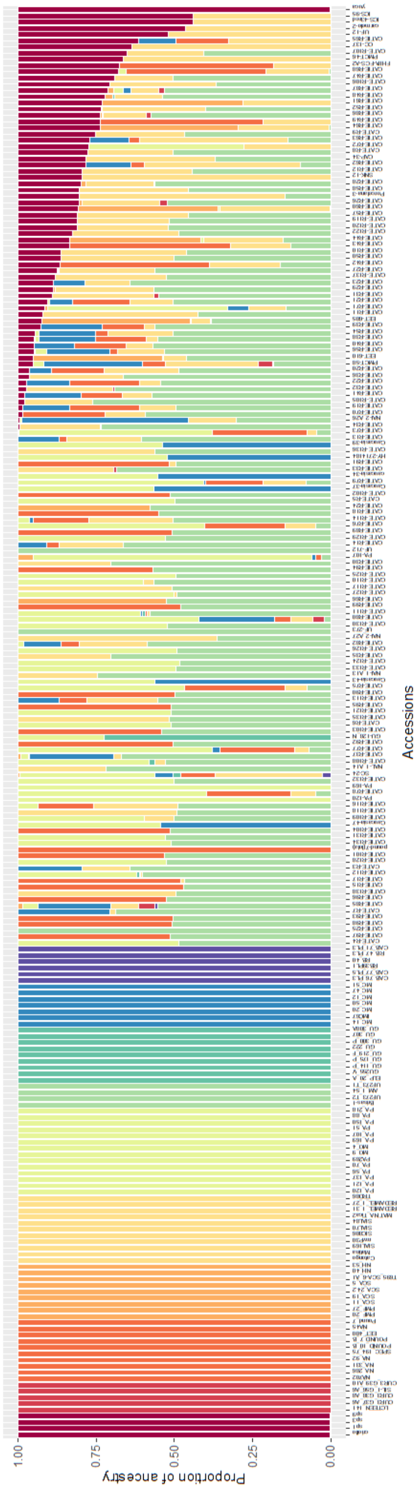

Supplementary Figure 11 | Ancestry assignment (K=10) for original ten clusters and newly genotyped admixed individuals. The order of the groups corresponds to the assignment in Figure 5 of the main manuscript. From left to right, the colors correspond to the following groups: Criollo (dark red), Curaray (red), Nanay (dark orange), Contamana (orange), Amelonado (light orange), Marañon (light green), Nacional (green), Guianna (dark green), Iquitos (blue) and Purus (purple). The assignment of newly

genotyped individuals to each ancestry allowed us to study the relationship of Criollo ancestry and the accumulation of deleterious mutations.

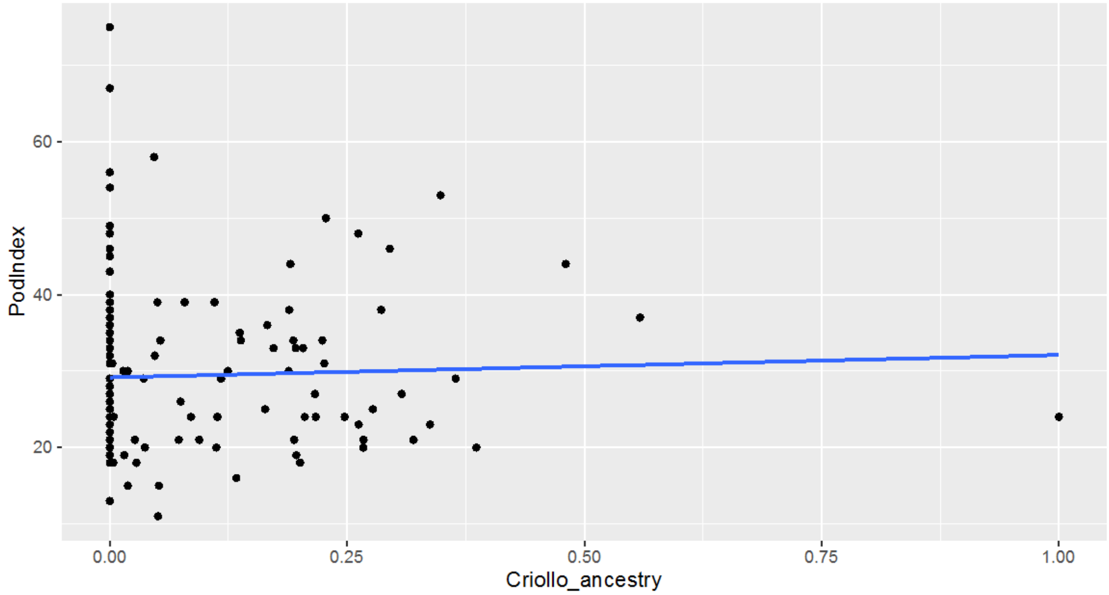

Supplementary Figure 12 | We found no association between the proportion of Criollo ancestry and the Pod Index

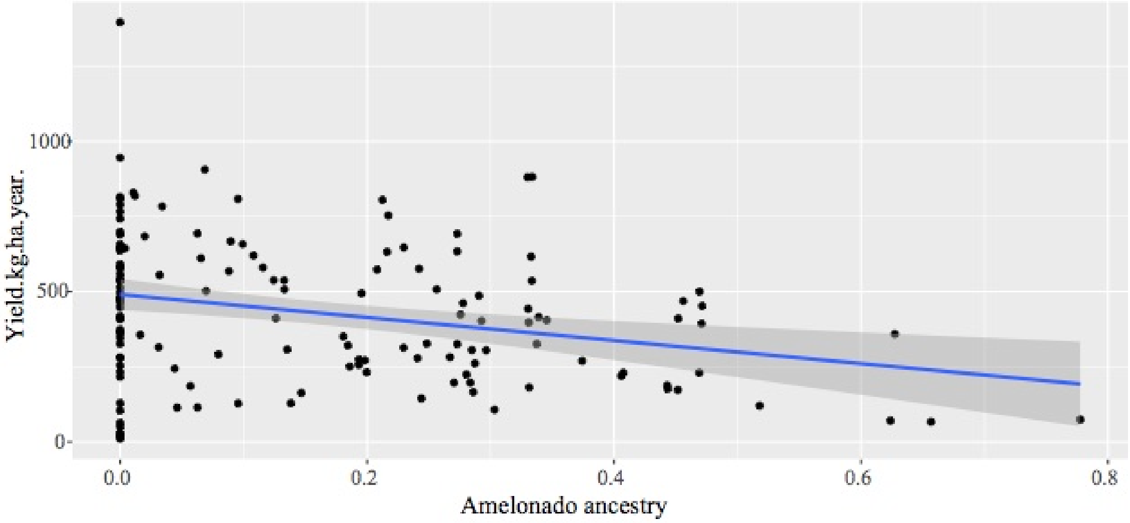

Supplementary Figure 13 | Association between Amelonado ancestry and Productivity (fitness)

Supplementary Tables

Supplementary Table 1 | List of accessions sequenced in this study. Sample source (collection of origin), Tree ID (when available), and research group are included.

Accession ID	*Sample source*	*Tree ID*	*Group*
2076	Ecuador		Indiana
2126	Ecuador		Indiana
2367	Ecuador		Indiana
2416	Ecuador		Indiana
2462	Ecuador		Indiana
2699	Ecuador		Indiana
2748	Ecuador		Indiana
AGU_3339_12	Trinidad	18276	Stanford
AMAZ-11_G18_A1	Ecuador		Indiana
AMAZ-11_G21_A10	Ecuador		Indiana
AMAZ-14_G23_A1	Ecuador		Indiana
AMAZ-14_G24_A5	Ecuador		Indiana
AMAZ_12	Trinidad	17443	Stanford
AMAZ_15_15	Trinidad	17421	Stanford
AM_1_54	Costa Rica		Stanford
AM_2_18	Costa Rica		Stanford
BE_10	Costa Rica	17422	Stanford
BR25	Indonesia (gDNA USDA)		Stanford
B_6_3	Trinidad	Tc17477	Stanford
B_6_8	Trinidad	18277	Stanford
Brisas-1	(Ecuador) gDNA USDA	Tc02491	Stanford
CAB_71_PL3	gDNA Miami	Tc02565	Stanford
CAB_76_PL3	gDNA Miami	Tc02567	Stanford
CAB_77_PL5	gDNA Miami	Tc02568	Stanford
CATIE_1000	Costa Rica	17423	Stanford
CCAT1119_EET544_A1	Ecuador		Indiana
CCAT1858_EET547	Ecuador		Indiana
CCAT4675_EET575	Ecuador		Indiana
CCAT4688_EET576_A1	Ecuador		Indiana
CCAT4998_EET577	Ecuador		Indiana
CCN10	Costa Rica	17446	Stanford
CCN51	Brazil, MARS		Indiana
CC_71	Costa Rica		Stanford
CL_27_50	Trinidad	Tc17482	Stanford
COCA_3370_5	Costa Rica	Tc04611	Stanford

CRUZ_7_14	Costa Rica		Stanford
CRU_101	Trinidad	Tc17492	Stanford
CRU_59	Trinidad		Stanford
CRU_89	Trinidad	Tc17488	Stanford
CS_146	Costa Rica	11453	Stanford
CUR3_G35_A1	Ecuador		Indiana
CUR3_G37_A6	Ecuador		Indiana
CUR3_G38_A8	Ecuador		Indiana
CUR3_G39_A10	Ecuador		Indiana
Catongo	Costa Rica	17445	Stanford
EB-19-1S	(Ecuador) gDNA USDA		Stanford
EB2237_A1	Ecuador		Indiana
EET103_Borde	Ecuador	17424	Indiana
EET397	Costa Rica	17447	Stanford
EET400_Arbol-122	Ecuador	Tc21291	Indiana
EET446_A1	Ecuador		Indiana
EET451_A1	Ecuador		Indiana
EET462_A1	Ecuador		Indiana
EET58_A1	Ecuador		Indiana
EET95_A2	Ecuador		Indiana
EET_103	Ecuador	17424	Indiana
EET_395	Trinidad	18186	Stanford
EET_400	Costa Rica	Tc17439	Stanford
EET_58	Trinidad	Tc17727	Stanford
EET_59	Trinidad	18302	Stanford
ELP_20_A	Costa Rica	Tc02198	Stanford
FSC_13	Costa Rica	18315	Stanford
GU255_V	Costa Rica	17425	Stanford
GU_114_P	Trinidad	Tc17459	Stanford
GU_175_P	Trinidad	Tc17460	Stanford
GU_291_F	Trinidad		Stanford
GU_222	Trinidad		Stanford
GU_300_P	Trinidad	Tc17463	Stanford
GU_307	Costa Rica	17426	Stanford
GU_308A	Costa Rica	Tc02175	Stanford
ICA_70	Trinidad	18321	Stanford
ICS39	Costa Rica	17448	Stanford
ICS40	Costa Rica	17449	Stanford
ICS_1	leaf from Miami		Stanford
ICS_43	Trinidad	18331	Stanford

ICS_6	Trinidad	Tc00551	Stanford
ICS_60	Trinidad	Tc18242	Stanford
ICS_61	Trinidad		Stanford
ICS_95	gDNA Miami	Tc16546	Stanford
IMC67	Trinidad	Tc17736	Stanford
IMC_12	Trinidad	18339	Stanford
IMC_14	Trinidad	17733	Stanford
IMC_20	Trinidad	Tc00707	Stanford
IMC_36	Trinidad	Tc00709	Stanford
IMC_47	Costa Rica	Tc16547	Stanford
IMC_50	Trinidad	Tc00560	Stanford
IMC_51	Trinidad	Tc00753	Stanford
JA_5_35	Trinidad		Stanford
JA_5_36	Trinidad		Stanford
JA_5_5	Trinidad		Stanford
K82	Papua New Guinea		Indiana
KA2	Papua New Guinea		Indiana
LCT46_A1	Ecuador		Indiana
LCTEEN_141	gDNA Miami	Tc01453	Stanford
LCT_EEN_46	Trinidad	Tc01431	Stanford
LCT_EEN_83_S-8	Trinidad		Stanford
LP_3_40	Trinidad	18348	Stanford
LP_4_48	Trinidad		Stanford
LX_32	Costa Rica		Stanford
LX_43	Costa Rica		Stanford
M01	Indonesia (gDNA USDA)		Stanford
M02	Indonesia (gDNA USDA)		Stanford
M04	Indonesia (gDNA USDA)		Stanford
M05	Indonesia (gDNA USDA)		Stanford
M06	Indonesia (gDNA USDA)		Stanford
M07	Indonesia (gDNA USDA)		Stanford
MAN_15_2	Costa Rica	17428	Stanford
MATINA_Tica2	Ecuador		Indiana
MO_109	Trinidad	Tc18213	Stanford
MO_4	Trinidad	18362	Stanford
MO_9	Trinidad	18370	Stanford
MO_99	Trinidad	Tc17508	Stanford
MXC_67	Trinidad	18391	Stanford
M_8	Trinidad		Stanford
Matina	Costa Rica		Indiana

Mocorongo	Costa Rica	17429	Stanford
NA45	Costa Rica	Tc00602	Stanford
NA702	Trinidad	TC0930	Stanford
NA_286	Trinidad	18395	Stanford
NA_331	Trinidad	Tc00923	Stanford
NA_33_T2	Trinidad	Tc00797	Stanford
NA_712	Trinidad	Tc0629	Stanford
NA_92	Trinidad	Tc00657	Stanford
NH_40	Bolivia, gDNA from Miami	6451	Stanford
NH_53	Bolivia, gDNA from Miami	6464	Stanford
OC_61	Trinidad	Tc17475	Stanford
PA107_A1	Trinidad		Stanford
PA289	Trinidad	Tc00511	Stanford
PA_107	Trinidad	18407	Stanford
PA_120	Trinidad	Tc18221	Stanford
PA_121	Costa Rica, gDNA from Miami	Tc00955	Stanford
PA_13	gDNA Miami	Tc18218	Stanford
PA_137	Trinidad	Tc15958	Stanford
PA_150	Trinidad	TC00501	Stanford
PA_169	Trinidad	Tc15974	Stanford
PA_218	Trinidad	18192	Stanford
PA_51	Miami	Tc00686	Stanford
PA_56	Trinidad	18396	Stanford
PA_70	gDNA Miami	Tc15954	Stanford
PA_88	gDNA Miami	TC00983	Stanford
PBC123	Indonesia (gDNA USDA)		Stanford
PMF_20	gDNA Miami	Tc11280	Stanford
PMF_27	gDNA Miami	11287	Stanford
POUND_10_B	Trinidad	Tc00858	Stanford
POUND_7_B	Trinidad	18419	Stanford
PlayaAlta1	Trinidad	Tc16545	Stanford
Pound_7	Costa Rica		Stanford
RB39PL1	Costa Rica	Tc02522	Stanford
RB_40	Costa Rica	Tc00449	Stanford
RB_47_PL3	Costa Rica	Tc02518	Stanford
REDAMEL_1_27	Trinidad	18180	Stanford
REDAMEL_1_31	Trinidad		Stanford
*SCA6***	gDNA Miami	Tc16548	Stanford
SCA_10	Trinidad	Tc00984	Stanford
SCA_11	Trinidad	Tc00882	Stanford

SCA_19	Costa Rica	Tc00522	Stanford
SCA_24.2	Costa Rica	Tc00523	Stanford
SCA_5	gDNA Miami	Tc00884	Stanford
SC_1	Costa Rica		Stanford
SIAL169	Costa Rica	Tc00179	Stanford
SIAL70	Costa Rica	Tc00185	Stanford
SIAL84	Costa Rica	Tc00186	Stanford
SIC806	Costa Rica	Tc00200	Stanford
SIL-1_G56_A6	Costa Rica		Stanford
SJ_2_22	Trinidad	18442	Stanford
SLC_4	Trinidad	Tc17513	Stanford
SNA0707	Costa Rica		Stanford
SPA_7	Costa Rica		Stanford
SPEC_194_75	Trinidad	Tc18235	Stanford
SPEC_54_1	Costa Rica	Tc05194	Stanford
T675_A645_A1	Ecuador		Stanford
T678_B60_A1	Ecuador		Stanford
T680_A1	Ecuador		Stanford
T682_A1_D147	Ecuador		Stanford
T684_EET233_A1	Ecuador		Stanford
T685_EET387_A1	Ecuador		Stanford
T686_LCT-368_A1	Ecuador		Stanford
T695_SCA-6_A1	Ecuador		Stanford
TAP10_G12_A1	Ecuador		Stanford
TAP3_G70_A2	Ecuador		Stanford
TAP6_G12_A1	Ecuador		Stanford
TIP-1_G41_A1	Ecuador		Stanford
TRD86	Trinidad		Stanford
TRD_45	Trinidad	18443	Stanford
TSA654Zymo	Costa Rica	17457	Stanford
TSH1188	Brazil, MARS		Stanford
TSH516	Costa Rica	17455	Stanford
UF12	Costa Rica	17434	Stanford
UF273_T1	Costa Rica		Stanford
UF273_T2	Costa Rica		Stanford
UF_11	Costa Rica	18446	Stanford
UF_668	Costa Rica	17456	Stanford
UF_676	Costa Rica	Tc13037	Stanford
UNAP2_G78_A2	Ecuador		Stanford
criollo	Costa Rica		Indiana

mvP30	Indonesia	Indiana
mvT85	Indonesia	Indiana
sp1	Venezuela (gDNA USDA)	Stanford
sp3	Venezuela (gDNA USDA)	Stanford
sp9	Venezuela (gDNA USDA)	Stanford

Sample marked with a ** is an offset from what SCA6 should be. It resulted in an admixed individual and researchers interested in looking at SCA6 should not use this accession as a representative sample from SCA6 (admixture analysis showed this is a hybrid).

Supplementary Table 2 | Annotation of polymorphic sites in the cacao genome.

Type of change	*chr_1*	*chr_2*	*chr_3*	*chr_4*	*chr_5*	*chr_6*	*chr_7*	*chr_8*	*chr_9*	*chr_10*	*Total*
splice_acceptor_variant	1137	1091	1077	1069	1155	1036	1041	941	1109	1006	10662
splice_donor_variant	1041	1065	1018	1011	1030	966	995	925	1034	977	10062
start_lost	881	857	848	870	870	819	836	806	862	821	8470
stop_gained	1852	1819	1676	1759	1950	1470	1727	1353	1704	1646	16956
stop_lost	888	865	870	862	888	853	828	829	871	834	8588
missense_variant	40756	36789	32090	35310	39172	27462	27189	21986	35056	26465	322275
*reg_region_ablation**	757	757	757	757	757	757	757	757	757	757	7570
splice_region_variant	6664	5877	5253	5278	5695	4573	3731	3707	5920	3863	50561
stop_retained_variant	826	813	800	807	815	806	795	789	804	794	8049
synonymous_variant	28976	25397	22197	23849	26200	19084	16520	15803	25544	16473	220043
3_prime_UTR_variant	35039	31120	27554	27252	29125	21817	17428	18657	31491	18345	257828
5_prime_UTR_variant	23013	19428	17491	17385	18449	14067	10813	11713	19689	11506	163554
*downstream_g_variant***	132861	132687	112431	112718	136761	91290	92960	72961	137247	96219	1118135
intergenic_region	301809	446024	298491	298152	434813	230026	234133	167223	406066	277742	3094479
intron_variant	92905	113223	105025	94243	119196	101995	122671	100143	116546	100909	1066856
upstream_gene_variant	321800	296212	243510	248306	286108	195094	170662	159877	297913	184225	2403707

* regulatory region ablation. ** downstream gene variant

Supplementary Table 3 | Coefficients GLM model adjusted to explain the differences in genetic diversity by group.

Coefficients	Estimate	Std._Error	t_value	Pr(>|t|)
(Intercept)	-7.457655	0.002082	-3582.4	<2e-16***
Contamana	1.544563	0.002707	570.6	<2e-16***
Criollo	0.189763	0.003874	48.99	<2e-16***
Curaray	1.427195	0.002825	505.26	<2e-16***
Guianna	0.572881	0.002931	195.5	<2e-16***
Iquitos	1.317511	0.002728	482.9	<2e-16***
Maranon	1.0866	0.002732	397.8	<2e-16***
Nacional	1.28207	0.002754	465.6	<2e-16***
Nanay	0.537165	0.002784	192.9	<2e-16***
Purus	1.210039	0.002731	443	<2e-16***
Admixed	1.614775	0.002774	582.1	<2e-16***

Supplementary Table 4 | Analysis of Variance Table

Response:-PC2	Df	Sum_Sq	Mean_Sq	F_value	Pr(>F)
pi	1	0.036303	0.036303	9.3134	0.01578*
Residuals	8	0.031183	0.003898		

Supplementary Table 5 | Coefficients GLM model explaining differences in the rate of accumulation of deleterious mutations between Amelonado and Criollo.

Coefficients	*Estimate*	*Std._Error*	*z_value*	*Pr(>|z|)*
(Intercept)	8.66615	0.01236	701.006	<2e-16***
ImpactTOLERATED	1.24366	0.01377	90.306	<2e-16***
PopulationCriollo	-1.76858	0.01627	-108.714	<2e-16***
freq_class(0.25,0.375]	-2.28433	0.03594	-63.567	<2e-16***
freq_class(0.375,0.5]	-2.59115	0.03772	-68.702	<2e-16***
ImpactTOLERATED:freq_class(0.25,0.375]	0.01512	0.03808	0.397	0.691
ImpactTOLERATED:freq_class(0.375,0.5]	-0.03374	0.03794	-0.889	0.374
PopulationCriollo:freq_class(0.25,0.375]	1.44586	0.034	42.531	<2e-16***
PopulationCriollo:freq_class(0.375,0.5]	2.0974	0.03425	61.236	<2e-16***

Supplementary Methods

Collection and DNA preparation for sequencing

We have constructed the first multi-population genomic variability dataset for cacao by analyzing the genomes of 200 accessions, selected from major collections of cacao. These samples represent a comprehensive collection from various geographical origins (genetic cluster), domestic accessions of common use in worldwide crops, and wild admixed individuals. Supplementary Table 1 contains the Accession IDs and the Tree ID of collected trees (when available) as well as the sample source where the leaf samples were obtained. USDA approval was given for the import of leaf material for DNA extraction and library preparation.

Annotation Data Analysis

The number of changes belonging to the main 16 categories is presented in Supplementary Table 2 and main Figure 1. As discussed in the main text, synonymous and especially missense variants are relevant because they were used to annotate the potential impact of these mutations in protein function. Splice acceptor (Splice_acceptor) and splice donor (Splice_donor) variants are relevant to potentially understand the underlying polymorphism in the variation for the number of differentially spliced transcripts in the species and among populations. The small number of start lost variants is also indicative of high conservation in the expression among genes or the changes in the use of potential codons for the start of translation of protein products. Perhaps even more interesting is the relatively higher number of stop gains observed in the analysis. Most of the stop gains (> 60%) seem to be located towards the end of the genes, which suggest that their effect in preventing the appropriate generation of proteins is rather limited and their negative effects will be minimal on average.

It is not surprising that most polymorphism is found in intergenic regions or generally annotated as upstream or downstream of genes. After non-coding variants found in intergenic regions, SNPs found in intronic regions are the most prevalent. Changes in intronic regions can often be neutral, from a functional perspective, but could contribute to differential lifespan of mRNAs, differential processing of the immature RNA including regulation of nonsense-mediated decay[1]. There is an increase in intron variant research because it has long been recognized that introns might be involved in mRNA transport or chromatin assembly[2,3]; and we expect that the variants identified in the genome will facilitate further work involving post-transcriptional regulation of expression.

Under a standard neutral model (with no changes in effective population size, drift and mutation balance) both statistics should converge to the same measurement (the basis for Tajima's D[4]). Overall, there is agreement between the two measurements of genetic diversity, with a slight underestimation of genetic polymorphism under θ_w (Supplementary Figure 1). The orthogonal comparison between our SNP data and a 6K SNPs chip specifically designed for cacao on selected accessions (CCN51, TSH1188, Pound7 and UF273_T1/T2) was 99.9% concordant.

Population Structure in **Theobroma cacao**

We used a ADMIXTURE[5] to estimate the number of underlying populations in *T. cacao*. Following this approximation, we concluded that the global LL maximum was reached in runs at

K=2 to *K*=15 (at least). *ADMIXTURE* includes a cross-validation (CV) procedure to help choose the "best" *K*, which is defined as the *K* that has the best predictive accuracy. Our analysis suggests that using cross-validation it is not possible to distinguish between K=10, K=11 and K=12. Although K=10 and K=11 seem to be more likely (better likelihood scores examined using Akaike information criteria).

In addition to the performed analyses we inferred a Neighbor-Joining phylogenetic tree using p-distances for chromosome 1. For this, we employed VCF Kit from the Andersen lab (https://github.com/Andersenlab/vcf-kit). The obtained tree was edited with FigTree (http://tree.bio.ed.ac.uk/software/figtree/) from the Rambaud's lab. The obtained phylogenetic tree recapitulates the same relationships observed with other methods that disentangled the underlying population genetic structure for the 10 populations (Supplementary Figure 3).

Distribution of genetic variation among genetic groups.

We tested for significant differences in genetic diversity across populations using a Generalized linear model assumed a Gaussian family against the log value of the genetic diversity using a model of the form: $\log(Y) = \beta_0 + \beta_i + \epsilon$ where *i* corresponds to the population or genetic group (Amelonado, Admixed, Contamanta, etc).

Theobroma cacao *differentiation along the West to East axis in the Amazon basin.*

We fitted a model (described in Figure 1C of the main manuscript) to explain the differences in genetic diversity along the Pacific/Atlantic axis of genetic differentiation captured in the second component of a multidimensional scaling. For this, we estimated the centroids for PC1 and PC2 of the data presented in Figure 1B (main manuscript). These centroids were used as predictors (β_i) to explain the differences in mean genetic diversity per population (measured as π, Y in the following model) under a simple linear model with a Gaussian family ($Y = \beta_0 + \beta_i + \epsilon$). Admixed individuals were excluded from the analysis.

Model-based analysis of population differentiation

We analyzed the history of population differentiation with TreeMix[15]. This program allows to estimate the evolutionary history of populations by modelling how the shared genetic variation and drift plays a role in determining the genetic relationships between populations. It allows to explicitly model how genetic variants along the genome drift and extend those models to explicitly include migration and how it contributes to the drift genetic components. For the analyses with TreeMix we used only intergenic regions. We used our annotation of the reference Matina genome to create bed files with intervals corresponding to the intergenic regions of the genome and extracted SNPs in these regions for our estimations. Bed files can be made available upon request.

Demographic history in **Theobroma cacao**

We modeled the distribution of variation within genomes to provide insights about the history and demography of ancestral populations with PSMC[17]. PSMC uses the distribution of heterozygote sites throughout the genome to estimate the time to the most recent common ancestor of a segment of sequence. For this, we first phased the genetic information of genomes

belonging to each one of the 10 populations characterized in cacao using ShapeIT[18,19]. Then, individual genomes were used to infer changes in demographic history for each population. We then combined the inferred history from multiple individuals from the same population and estimated smoothed PSMC curves per populations (shown in main manuscript Figure 2).

In addition to PSMC analyses we performed demographic analyses using SMC++, a method that can leverage information from multiple individuals from the population (as opposed to PSMC) to infer population size changes in the past[25].

Detailed demographic analysis of Cacao domestication
To further understand the population demographic history during the process of domestication we used a different approximation and built a demographic model based on the observations from the results of the PSMC and TreeMix analysis. The results from TreeMix suggested that Criollo and Curaray are the most related populations and the results from PSMC showed that both populations have been declining over time. We examined three alternative models: i) a simple model of isolation without migration (model A); ii) a model of isolation with migration (model B); and iii) a modified model of isolation with migration in which we allow the ancestral population prior to the split to be changing in time and the populations post split to change in time (model C). For each model, we estimated the corresponding likelihood and compared the relative fit of the models using Akaike information criteria.

LD decay in the populations of *Theobroma cacao*
We estimated pairwise LD per population using vcftools[19] using the D' statistic. In order to estimate the LD decay as a function of genetic physical distance we fitted a non-linear model assuming a recombination rate in the same order of magnitude as the mutation rate following the non-linear approximation proposed by Abecasis et al[38].

Effects of historical Population Size on Inbreeding
We compared the levels of inbreeding among populations estimated as F statistics[27,28], using an ANOVA. We used the admixed cluster of individuals as a baseline to compare the naturally defined genetic groups (main Figure 4A). The reason for this is that recent admixed individuals were not expected to have significant inbreeding and consistently showed small F values.

Genes in genomic regions under selection
We used XP-CLR explained in the main methods section) to identrify regions of the genome that are inconsistent with the underlying demogrpahics that explains the split between Criollo and Curaray populations. We also identified the top 1% of differentiated regions of the genome using Fst, estimated in windows of 1Kb.

Accumulation of deleterious mutations
In using Sorting Intolerant from Tolerant (SIFT) 4G[40] we annotated non-synonymous (replacement) mutations for further analysis. Each prediction also provides a SIFT median score which measures the diversity of the sequences used for prediction. The SIFT median score ranges from 0 to 4.32, ideally it would be between 2.75 and 3.5. A warning with low confidence

occurs when the SIFT median score is greater than 3.25 because this indicates that the prediction was based on closely related sequences. The low confidence in SIFT score means that the protein alignment does not have enough sequence diversity because the position artificially appears to be conserved, an amino acid substitution may incorrectly predicted to be damaging. This score system was used to support the assignment of replacement substitutions as deleterious or tolerated for the rest of the analyses.

Prior to a Mantel-Haenszel test for specific effects, we fitted a generalized linear model to the count data for deleterious/tolerated mutations in Amelonado and Criollo, assuming a log-linear model. This model allowed us to test for general trends in the data and show that there is a significant difference in the number of deleterious mutations among Criollo and Amelonado along binned classes of minor allele frequency. Because we have differences in sample size between Criollo and Amelonado, we could not compare directly all the minor allele frequency classes and decided to bin them, making the direct comparison feasible. For each allele frequency class: rare (0-0.25], intermediate (0.25-0.375] and frequent (0.375,0.5] the number of predicted deleterious and tolerated mutations were identified using SIFT4G. Our model of the form:

$$Y_{ij} = I_i | \, freq + Pop_j \, | freq + \varepsilon_{ij},$$

was set to explain the counts of mutations as a function of their impact (deleterious/tolerated) and the population of origin, taking into account that different minor allele frequency classes will have different absolute counts in them. In our model Y_{ij} are the counts of the number of SNPs, I_{ij} corresponds to the impact (deleterious vs tolerated mutations) and Pop_i corresponds to the population (Amelonado and Criollo) and the comparisons are done conditional on frequency class bin of minor alleles. The link function is assumed to be Poisson.

Association between Criollo ancestry and productivity.
We show that Criollo populations sustain deleterious mutations at a higher frequency than Amelonado, even though both populations present a high frequency of self-compatible individuals. It remained to be tested what was the phenotypic effect of the proportional increased accumulation of deleterious mutations in the Criollo populations. For this, we used an independent dataset of plants for which productivity (measured as yield of beans per hectare per year) had been measured. We genotyped these plants with a Fluidigm array developed based on SNPs that were generated from some of the samples from the 200 genomes[44].

After genotyping, we merged the SNPs from newly genotyped individuals with SNPs from the individuals clearly assigned to each one of the 10 populations. The intersected dataset resulted in 7,621 SNPs. We then used *ADMIXTURE*[5] using a supervised assignment mode to estimate the proportion of ancestries to each one of the 10 populations. For each individual in the newly genotyped dataset, we also used vcftools[45] to estimate inbreeding coefficients. The proportion of ancestry assigned to each one of the newly genotyped individuals can be seen in Supplementary Figure 11.

Following the estimation of ancestry, we estimated if the proportion of Criollo ancestry is associated with a reduction in the productivity using a simple linear model, while controlling for

inbreeding. We built a generalized linear model assuming a Gaussian family of the form:

$Y = \beta_0 + \beta_1 + \beta_2 + \varepsilon$, where Y corresponds to the yield, β_0 corresponds to the intersect, β_1 corresponds to the proportion of Criollo ancestry and β_2 is the coefficient of inbreeding F, estimated for each individual.

We compared the model that considers inbreeding and a reduced version $Y = \beta_0 + \beta_1 + \varepsilon$ that considers only the proportion of Criollo ancestry.

Supplementary Results and Discussion

Population Genetic Structure in *T. cacao*

Initial genetic analyses, with microsatellites markers, have uncovered a large number of genetic groups and clear differentiation between the trees found in the Amazon basin and the Criollo varieties found in Central America[6]. This work helped characterize cacao germplasm into 10 major genetically differentiated groups: Amelonado, Contamana, Criollo, Curaray, Guianna, Iquitos, Marañon, Nacional, Nanay and Purús[6]. Additional analyses performed with microsatellites suggested that Criollo, the most likely representative of the cacao domesticated in Mesoamerica, is more closely related to trees from the Colombia-Ecuador border than trees from other South American groups[6]. Yet, there is a huge gap in our understanding of genomic variation in the species which makes it difficult to propose clear scenarios for the evolution of natural populations and the domestication of *T. cacao* and how this might be exploited by the agronomist for crop improvement and sustainability. The assignment of ancestries to K=10 is easy to interpret using previous work that has characterized the genetic variation of *T. cacao* with microsatellite markers and proposed 10 main populations or genetic groups to explain genetic differentiation in the species[6]. The assignment based on K=10 or K=11 produce overall similar results. Yet, K=11 reveals further population structure (which has been observed in previous analyses and sometimes reported as substructure[6], which could be particulary important to understand the genetic ancestry of the hybrids (wild and domesticated). Based on our analyses, we designated five arbitrary groups of admixed individuals based on the major contribution of genetic clusters to mixed ancestry (Figure 1A of the main manuscript, left to right). The identification of individual samples and their assigned ancestry is provided in Supplementary Figure 2. Their position of individuals in the plot is the same shown in the figure in the main document. Group I (horizontal red bar above plot) is characterized by a declining gradient in the contribution of Criollo ancestry, with a large majority of individuals presenting Criollo/Amelonado ancestry and a wide number of accessions presenting complex patterns of admixture that includes contributions from Nacional, Iquitos and Purus. Group II (horizontal yellow bar) is defined by a gradient of Curaray ancestry with major contributions from Nacional and Amelonado. Group III (horizontal green bar) is defined by a gradient of Nanay ancestry with major contributions from Iquito ancestry. Group IV (horizontal blue bar) is defined by a gradient in Contamana ancestry and presents major contribution from Iquitos and Nacional. Group V (horizontal purple bar) is defined by a relatively equivalent contribution of Amelonado and Nacional ancestry. There is evidence for additional substructure among these groups, not previously identified but consistent with observations of other genetic groups[7,8]. More specifically, we identify an additional component of ancestry that results from the decomposition of the Amelonado group into two clusters of ancestry. We believe that this second component of Amelonado ancestry is real, even though only a single individual can be fully assigned to it, because it is found to be the major contributor to the ancestry of Group V of admixed individuals. These results are noteworthy because most cultivated varieties seem to contain a large component of Criollo and Amelonado ancestry which means there ample genetic potential in this critically important crop for the plant breeder to exploit.

Although the discriminative analyses performed with *ADMIXTURE* provide a good approximation to identify the underlying number of populations (or components of ancestry in a population of admixed individuals), it does not provide an intuitive way to interpret the

relatedness among populations. In order to gain a better understanding of the population structure in *T. cacao* we performed a multi-dimensional scaling analysis on the same set of SNPs employed for the *ADMIXTURE* analysis. We recapitulated the separation of groups observed in the *ADMIXTURE* analysis, and the MDS analysis added information about the relative differentiation between populations and the contributions of the different groups to the admixed individuals (See Figure 1 of main manuscript).

Distribution of genetic variation among genetic groups.

For convenience, we present the assessment of the distribution of genetic diversity genome-wide per population and across the genome per population, using the number of pairwise differences per site (π). Our analyses reveal that there are remarkable differences in the magnitude of genetic variation among populations of cacao. We used a generalized linear model to compare the genetic diversity among groups. Our Generalized linear model assumed a Gaussian family against the log value of the genetic diversity using a model of the form: $\log(Y) = \beta_0 + \beta_i + \epsilon$ where *i* corresponds to the population or genetic group (Amelonado, Admixed, Contamanta, etc).

```
log.glm <- glm(log(PI) ~ Group, family=gaussian, data=data)

Deviance Residuals:
    Min       1Q    Median       3Q      Max
-5.8607  -0.6223   0.0838   0.6870   3.7276
```

Our results clearly show that belonging to a genetic group modifies considerably the expectation of the levels of genetic diversity observed in a sample, when compared to the Amelonado population (Supplementary Table 3). We used Amelonado to compare against as it presents small levels of variation overall and because the reference genome employed (Matina) is an individual of Amelonado ancestry. It is remarkable that Criollo presents the largest impact towards reducing the expected genetic diversity. This is consistent with our results that Criollo populations present a very reduced effective population size, probably the result of a very strong and relatively recent domestication event. Not surprisingly, admixed individuals present the largest positive effect towards the increase on genetic diversity, but this effect is very similar to that observed of the estimates for the Contamana group which, to the effects of this work, is considered a wild population.

The differences in genetic diversity across populations is partially explained by differences in demographic history experienced by each population and partially by the differences in the rates of selfing and outcrossing experienced by each population. Individuals. Differences in the proportion of self-compatible and self-incompatible individuals in each population modifies the rate of selfing experienced in each population, which in turn has a strong impact on the magnitude of genetic diversity maintained in the population[9,10].

Our analyses also reveal that the distribution of genetic diversity along the genome of *T. cacao* presents a heavy tail, similar to what has been observed in other organisms[11,12] (Supplementary Figure 4). Differences in the distribution of genetic polymorphism along the genome have been interpreted, under population genetic premises and appropriate models, as

corresponding differences in the effective population size along the genome[11-14], which could then affect the rate of adaptive molecular evolution in eukaryotes[13]. Areas of the genome with a larger effective population size could be more prone to fast adaptive evolution from standing variation than other regions of the genome and the differential distribution of genetic variation along the chromosomes in different genetic groups suggests that *T. cacao* harbors a large potential for adaptation from standing variation (Supplementary Figures 3 and 4). It can be seen in Supplementary Figure 4 that there are regions of the genome with considerably more variation than others, a pattern that requires further investigation. In the context of management of a domesticated species, this implies that potentially different regions of the genome are more amenable of artificial selection than others in different populations of *T. cacao*. Our analysis reveals that the process of domestication of the Criollo variety in Mesoamerica was the result of a single event, with no evidence of recent gene flow to this group from other genetic clusters. Also, we find no additional signatures for domestication as strong as the one found for the Criollo group in the rest of the genetic clusters. We show for the first time that the process of differentiation of the genetic clusters presents a complex pattern of historical admixture. Our analysis of genetic variation on the admixed individuals reveals that, despite the large number of well-differentiated populations in cacao, only a few ancestry components can be found in admixed individuals, suggesting there is a large amount of untapped genetic variation in the species.

The difference in overall genetic diversity across groups is likely due to the combined contribution of differences in effective population size and demographic histories, with Criollos showing the lowest genetic diversity (π=0.27%) and Contamanas, Nacional, and Admixed individuals presenting the highest diversity (π=0.32%, π=0.31%, π=0.37%, respectively, Supplementary Figure 3). We also identify a clear pattern of high heterogeneity in the distribution of genetic diversity along the genome suggesting differences in effective population size along the genome potentially driven by artificial and natural selection (Supplementary Figures 4 and 5).

Admixture and MDS analyses provide a good graphical representation of the genetic structure in *T. cacao*. In addition to the overall analysis of population differentiation using F_{st}s, we show that there are regions of the genome that are more differentiated when all pairs of populations are compared to each other. This pattern of differentiation suggests that not only genetic diversity, but also the genetic divergence between populations has distribution with long tails. We exploit this feature in the analysis of selection where we show that some regions of the genome present significant differences in the local two-dimensional site frequency spectrum when compared to the genome-wide site frequency spectrum described by the demographics. In additional figures available at (https://labs.wsu.edu/populationgenomics/resources/data_figures/) show the pairwise Fst along the genome for each population against the rest of the populations. In these figures the grey line represents the median F_{st} and the top red line the upper 95 % confidence interval. This is a pattern we examined in more detail between the separation of the Criollo and Curaray populations in the context of domestication.

Theobroma cacao ***differentiation along the West to East axis in the Amazon basin.***
Our analysis shows a significant association between geographic location (as described by genetic differentiation, Supplementary Table 4) and genetic diversity, with larger genetic

diversity available in groups closer to the Pacific end of the Amazon Basin (negative PC2 values) and a progressive reduction in genetic diversity towards the Atlantic.

Coefficients:
(Intercept) β_i
0.2015 -72.7140

Model-based analysis of population differentiation
We analyzed the history of population differentiation with TreeMix[15]. Two important results from this analysis are: i) that the domesticated Criollo populations have undergone a large amount of drift (larger than any other population) and ii) that all of the evidence of migration and admixture suggest that no additional contribution of any group has occurred after the domestication of *T. cacao* in Mesoamerica. The strongest evidence indicates a recent contribution of Iquitos to Nanay (red arrow in main Figure 2B), which is consistent with the partial ancestry of Iquitos identified in some of the individuals belonging to the Nanay group in the admixture analysis (main Figure 1A). Ancient admixture analyses, in the form of pairwise f_3 statistics [16], confirm that Criollo and Curaray are significantly closer to one another than to any other group and no evidence of significant admixture can be found.

***Demographic history in* Theobroma cacao**
The results across individuals were highly similar and smooth spline regression showed that all populations of cacao seemed to have undergone a population decline since the last glacial maximum. The Criollo populations have a much smaller population size, but we detected a similar trend in population reduction. For our estimation of the population sizes, we assumed a similar mutation rate to that observed in other plants (Arabidopsis) of 7.1×10^{-9} mutations per base pair per generation[20,21]. Recent work has suggested that mutation rates could be half of that estimated previously 3.1×10^{-9} [22], yet the authors find that this mutation rate is not different from previously estimated rates. For our detailed demographic analysis inferred from the site frequency spectrum we used and report results for both mutation rates. We also assumed a generation time (the time that it takes to go from seed to seed) as 5 years[23]. The choice of 5 years as a generation time is the result of the observation that it takes on average 5 years to go from seed to seed. We believe this is an appropriate selection of the generation time for the species. Although the general trend is towards the loss of genetic diversity, two different dynamics are evident. First, the Curaray populations and, to a lesser extent the Iquitos and Purus populations, show signatures of an initial increase in their population size followed by a decline. This pattern that could be explained by admixture as it has been observed in other organisms[24]; as well as real population increases and decreases in time. Second, we observe a much more recent and far smaller overall population size for the Criollo group which is consistent with the idea of a strong domestication event in recent times from a relatively small pool of individuals (see Figure 2D).

Our results of the SMC++ analysis showed similar overall results. The general trends in population size change, namely a decline that coincides with Last Glacial Maximum for most populations, is still observed, even if the general magnitude of the change is not the same (see main Figure 3E). Most of these methods perform poorly to infer recent demographic changes and

we maintain a certain skepticism in the estimated values for recent times when admixture may have occurred. In order to better address the reconstruction of the split between Curaray populations and Criollo we used methods that rely in the site frequency spectrum[26]. We used the results from PSMC/SMC++ as a general guide to presume that populations of Criollo and Curaray change in time, but we do not bounded the parameters indicating the direction of the change.

Effects of historical Population Size on Inbreeding

We observe an increase in the amount of inbreeding (estimated as F statistics [27,28]) when the admixed cluster of individuals is compared to the naturally defined genetic groups (main Figure 4A). The general trend shows an increase in inbreeding from Iquitos, Nacional, Curaray, Contamana, Marañon and Purus and even higher levels of inbreeding in Guianna, Criollo, Nanay, and Amelonado (main Figure 4A). Amelonado presents a much higher level of inbreeding, given what would be expected under its historical demographics. *T. cacao* shows a unique self-incompatibility mating system where some individuals in the species are self-incompatible (SI) while some other are self-compatible (SC) [29]. A reduced number of accessions in the species have been characterized for SC/SI, and there has not been a thorough assessment of the distribution of SC/SI in most genetic groups, so that the overall frequency of SI/SC in the species is largely unknown. Despite this, there is field evidence suggesting that the Amelonado population presents a high frequency of SC individuals[30]. Similarly, most plants in the Criollo group have also been described to be SC[23].

Detailed demographic analysis of Cacao domestication

We examined three alternative models: i) a simple model of isolation without migration (model A); ii) a model of isolation with migration (model B); and iii) a modified model of isolation with migration in which we allow the ancestral population prior to the split to be changing in time and the populations post-split to change in time (model C). The fitting to model A, isolation with no migration, was the worst model explain the data (LL = -15818.1, AIC =31642.2). The fitting to Model B, representing a simple isolation with migration model, was the second best fit (LL = -1251.61, AIC = 2513.22). The best fitted model is Model C, a change in the ancestral population size prior to split and also after the split (LL= -664.88, AIC=1345.76). The AIC values support Model C as the best fitting model. All estimations were performed masking the rare variants present as singletons in either populations or as doubletons in either or both populations. The reason for this is that Criollo and Curaray populations have a significant number of individuals able to self and selfing affects the coalescence by increasing the coalescence rate at the top of the genealogies[31,32] and we have observed via simulations that this can strongly impact the frequency of rare variants (namely singletons and doubletons). The figures for fitted model C in the main manuscript have blank boxes in the 2D-SFS representing this (Figure 2E).
Current dogma suggests cacao was introduced to Mesoamerica in Omec times from cacao varieties present in the Upper Amazon (Northern South America), the hotbed of diversity for the species[23,33]. Anthropological research, in particular, supports this view [23,34,35]. Another line of evidence suggests that the route of domestication of the chocolate tree could have dispersed throughout the Amazon Basin along two routes: one leading north and another leading west. According to this hypothesis, domestication of cacao would have occurred in South America and then spread to Central America and Mexico through Native American trade networks [36]. In addition to the interest in understanding the historical domestication of cacao, there is

tremendous agricultural knowledge need in assessing how development of land races and varietals as well as outcrossing to genetically diverse germplasm has shaped diversity in modern cacao crops [6,37]. The results from our models are consistent with the general idea that Cacao Criollo was domesticated in Mesoamerica, but more detailed information about the possible alternative routes will require additional genotyping of plants along the alternative spatial paths accompanied with appropriate analyses.

We re-estimated parameters under an isolation with migration model for each one of the bootstrapped datasets and we finally used the empirical cumulative distribution (ecd) for the parameters to estimate the 95% confidence interval as those values that fell within the 0.025 and 0.975 quantiles of the empirical distribution. Supplementary Figure 6 shows the ecd for the time since split estimation of the separation between Curaray and Criollo populations (left panel for mutation rates $7.1\text{x}10^{-9}$ and right panel for $3.1\text{x}10^{-9}$) and Supplementary Figure 7 shows the ecd for the fraction of the ancestral Curaray population that likely served as a seed for Criollo domestication. For our estimations we followed the same assumptions for the mutation rate (7.1×10^{-9} mutations per base pair per generation) [20,21] and generation time (5 years) [23].

We have also explored the impact of relaxing our assumptions about generation time in the species. Assuming a generation time of 15 years, the expected time of Divergence between Curaray and Criollo would be 10,861 years BP, and the confidence interval for the time of divergence between populations would be 95% CI = 7444.1 – 32708.2 years BP. Yet, anthropological evidence supports a timeline for the peopling of South America at around 13,000 years BP, with additional evidence suggesting that human settlements were able to develop major crops only 8,000 years BP. Estimates of divergence between populations much older than our expectation for the settling of the Americas are less likely.
We further explored other possible generation times of 50 or 70 years per generation and maximum likelihood estimations of divergence are more unlikely than those estimated under a 15 years per generation ($\text{time}_{50\text{ years gen}}$ = 36,203 years BP, $\text{time}_{70\text{ years per gen}}$ = 50,685 years BP).

A potential caveat of these analyses is the possibility that the likelihood surface of the explored models have ridges in which combinations of parameters like divergence time and migration produce estimates with comparable likelihood. We explore the validity of our model by examining the likelihood surface of the model for different combinations of parameters. Simulations and likelihood estimations were performed in dadi for a wide range of values of migration from Curaray to Criollo (m21), migration from Criollo to Curaray (m12) and time of divergence (T2). We first examined the likelihood profile for combinations of values of the two migration parameters (Supplementary Figure 8A). As it can be seen, for a reasonable ranges of values in Supplementary Figure 8A, no ridges are found and the distribution of likelihood values suggest that our ML estimates better describe the data. Next, we explored the likelihood surface of migration rates and time of divergence for each migration parameter. We find that there are no ridges in the case of migration from Curaray to Criollo and time of divergence (Supplementary Figure 8 B). Although a wider range of times of divergence are similarly likely for the most likely migration rates from Criollo to Curaray (Supplementary Figure 8C), the boundaries of the 3 times likelihood overlap well with our confidence intervals estimated from bootstrap. Given the combined likelihood surfaces from the combination of parameters we are confident that our estimates reflect well the information estimated from the data. Additional data will likely

improve the confidence interval around these estimates by combining the dadi analyses with recent analyses based on patterns of Identity by Descent sharing among individuals.

LD decay in the populations of *Theobroma cacao*
Supplementary Figure 6 shows the LD decay per population. As expected the Criollo population presented a much more extended elevated linkage than the other populations. Interestingly, Nanay populations also show elevated levels of linkage. Nacional, Contamana and Marañon showed much faster decay of LD as compare to other populations.

Genes in genomic regions under selection
The results of the analyses with XP-CLR[39] were analyzed prior to reporting regions under selection to intersect the windows which were in the vicinity to each other. We then identified putative genes using the current annotation for the Matina reference genome. Genes that overlapped with windows in which selection was detected are reported in Supplementary Data 1.

In addition to identifying regions of the genome under selection with XP-CLR we explore what regions of genome are more differentiated than the background by identifying those 5Kb windows that are in the top 1% of the Fst values for comparisons between Criollo and Curaray populations. We intersected the windows, part of the top 1% Fst outliers with the gene annotation. A list of genes identified in regions highly differentiated is provided in Supplementary Data 2.

Accumulation of deleterious mutations
Our model of the form:

$$Y_{ij} = I_i |\, freq + Pop_j \,| freq + \varepsilon_{ij},$$

was set to explain the counts of mutations as a function of their impact (deleterious/tolerated) and the population of origin, taking into account that different minor allele frequency classes will have different absolute counts in them. The values of fitted coefficients are shown in Supplementary Table 5 and Supplementary Figure 10 presents a graphical representation of the mosaic plot that better describes the results.

Everything else being equal, with similar selfing rates across populations, it is expected that Amelonados and Criollos will present remarkable differences in the accumulation of deleterious mutations as the result of the differences in the magnitude of the population size reductions and the impact of domestication in Criollo. We decided to use Amelonado as a contrasting group because we could control for the similar frequency of selfing in the populations.
Our analyses aimed at understanding the pattern of accumulation of deleterious mutations in populations with similar levels of inbreeding driven by selfing and yet very different domestication pressures produce a pattern that has been revealed in other systems like maize, rice and composite flowers [41-43]. The pattern we show in *T. cacao* is consistent with other observations. We extend the work to test how this reflects in the fitness (measured as productivity of seeds) in *T. cacao*, something that has not been tested in long lived arboreal crops like cacao. In the next section we show how the increase in proportion of Criollo ancestry and

thus the relative frequency of deleterious mutations impact the productivity, a proxy for measuring the reproductive component of fitness.

Association between Criollo ancestry and productivity.
Fitting of the full model suggests that as the proportion of Criollo increases, the reduction of yield is highly significant

Coefficients:

Intercept	Criollo_ancestry	F (inbreeding)
452.8	-555.2	-124.6

Degrees of Freedom: 145 Total (i.e. Null); 143 Residual
(4 observations deleted due to missingness)
Null Deviance: 8249000
Residual Deviance: 7420000
AIC: 2004

A likelihood ratio test suggests that the full model marginally explains the data better than the reduced model

Analysis of Deviance Table

Model 1: Yield.kg.ha.year. ~ Criollo_ancestry
Model 2: Yield.kg.ha.year. ~ Criollo_ancestry + F

Model	Resid	Df Resid.	Dev Df	Deviance	Pr(>Chi)
1	144	7565221			
2	143	7419722	1	145499	0.09402

The difference between the two models is not statistically significant. We also noticed that the size of the effect for the Criollo ancestry on productivity is at least 4.4 times larger than the size of the effect for inbreeding to explain the differences in yield. Taken together, we can say confidently that the proportion of Criollo ancestry, and thus the increase in higher frequency deleterious mutations, have a strong impact on productivity in cacao. Diagnostic plots for the fitting of the model are provided in Diagnostic1.zip. These results have a special appeal given that there is no appreciable association between Criollo ancestry and Pod Index (number of pods required for 1 kg of dried cocoa without testa). The lack of association between Criollo ancestry and Pod Index could be consistent with an interpretation in which the accumulation of deleterious mutations have decreased the fitness (kilograms of beans per hectare), but not the overall quality and ability to prepare chocolate from the cacao trees (Supplementary Figure 12). Yet, accurate analyses of quality would be necessary to test this proposition.

In addition to the analysis with the Criollo ancestry, we analyzed how the fitness (productivity) decays with the increase in Amelonado ancestry in the accessions. Similarly, we fitted a

generalized linear model assuming a Gaussian family to the Productivity as a function of Amelonado ancestry and found that the coefficient is equal to -383.1 (See Supplementary Figure 13), implying that the decay in productivity due to an increase in Criollo ancestry (555.2) is on average ~170 Kg per hectare per year larger than that due to Amelonado ancestry.

The results presented here focus on the analysis of productivity as a proxy for fitness, and it is important to demonstrate why this is a good proxy. Number of beans per pod has been shown to vary in cacao plants ranging from 20 to more than 50 beans [46]. Although Criollo cacao is not well represented in the International Cocoa Germplasm Database (http://www.icgd.rdg.ac.uk/), it is possible to extract information from all the Bean numbers per pod available for Amelonado. Based on 30 accessions (multiple observations from CATONGO, Matina, REDAMEL, SIAL, SIC and TRD), the mean number of beans per pod is 36.8 and a range from 30.4 to 42 pods per pod. Earlier work done in cacao Criollo has shown that 95% of pods contain from less than 20 to 30 beans with only a small fraction reaching numbers as high as 40 [47]. These results are consistent with the use of productivity with fitness, given that overall the number of beans per pod in Criollo tends to be lower than that of Amelonado.

Additional Figures not inserted in the document (Fst) are available in the website:
https://labs.wsu.edu/populationgenomics/resources/data_figures/

References

1 Jo, B. S. & Choi, S. S. Introns: The Functional Benefits of Introns in Genomes. *Genomics Inform* **13**, 112-118, doi:10.5808/GI.2015.13.4.112 (2015).

2 Rodrigues, J. P. *et al.* REF proteins mediate the export of spliced and unspliced mRNAs from the nucleus. *P Natl Acad Sci USA* **98**, 1030-1035 (2001).

3 Ryu, W. S. & Mertz, J. E. Simian virus 40 late transcripts lacking excisable intervening sequences are defective in both stability in the nucleus and transport to the cytoplasm. *Journal of Virology* **63**, 4386-4394 (1989).

4 Tajima, F. Statistical Method for Testing the Neutral Mutation Hypothesis by DNA Polymorphism. *Genetics* **123**, 585-595 (1989).

5 Alexander, D. H., Novembre, J. & Lange, K. Fast model-based estimation of ancestry in unrelated individuals. *Genome Res* **19**, 1655-1664, doi:10.1101/gr.094052.109 (2009).

6 Motamayor, J. C. *et al.* Geographic and genetic population differentiation of the Amazonian chocolate tree (Theobroma cacao L). *PLoS One* **3**, e3311, doi:10.1371/journal.pone.0003311 (2008).

7 Zhang, D. *et al.* Dissecting Genetic Structure in Farmer Selections of Theobroma Cacao in the Peruvian Amazon: Implications for on Farm Conservation and Rehabilitation. *Tropical Plant Biology* **4**, 106-116, doi:10.1007/s12042-010-9064-z (2011).

8 Zhang, D. *et al.* Genetic diversity and spatial structure in a new distinct Theobroma cacao L. population in Bolivia. *Genetic Resources and Crop Evolution* **59**, 239-252, doi:10.1007/s10722-011-9680-y (2011).

9 Charlesworth, D. & Charlesworth, B. Inbreeding Depression and Its Evolutionary Consequences. *Annu Rev Ecol Syst* **18**, 237-268, doi:DOI 10.1146/annurev.ecolsys.18.1.237 (1987).

10 Charlesworth, D., Morgan, M. T. & Charlesworth, B. Mutation Accumulation in Finite Outbreeding and Inbreeding Populations. *Genet Res* **61**, 39-56 (1993).

11 Piganeau, G. & Eyre-Walker, A. Evidence for variation in the effective population size of animal mitochondrial DNA. *PLoS One* **4**, e4396, doi:10.1371/journal.pone.0004396 (2009).

12 Cornejo, O. E., Fisher, D. & Escalante, A. A. Genome-wide patterns of genetic polymorphism and signatures of selection in Plasmodium vivax. *Genome Biol Evol* **7**, 106-119, doi:10.1093/gbe/evu267 (2014).

13 Gossmann, T. I., Keightley, P. D. & Eyre-Walker, A. The effect of variation in the effective population size on the rate of adaptive molecular evolution in eukaryotes. *Genome Biol Evol* **4**, 658-667, doi:10.1093/gbe/evs027 (2012).

14 Gossmann, T. I., Woolfit, M. & Eyre-Walker, A. Quantifying the variation in the effective population size within a genome. *Genetics* **189**, 1389-1402, doi:10.1534/genetics.111.132654 (2011).

15 Pickrell, J. K. & Pritchard, J. K. Inference of population splits and mixtures from genome-wide allele frequency data. *PLoS Genet* **8**, e1002967, doi:10.1371/journal.pgen.1002967 (2012).

16 Patterson, N. *et al.* Ancient admixture in human history. *Genetics* **192**, 1065-1093, doi:10.1534/genetics.112.145037 (2012).

17 Li, H. & Durbin, R. Inference of human population history from individual whole-genome sequences. *Nature* **475**, 493-496, doi:10.1038/nature10231 (2011).
18 Delaneau, O., Marchini, J. & Zagury, J. F. A linear complexity phasing method for thousands of genomes. *Nat Methods* **9**, 179-181, doi:10.1038/nmeth.1785 (2011).
19 Delaneau, O., Zagury, J. F. & Marchini, J. Improved whole-chromosome phasing for disease and population genetic studies. *Nat Methods* **10**, 5-6, doi:10.1038/nmeth.2307 (2013).
20 Lynch, M. Evolution of the mutation rate. *Trends Genet* **26**, 345-352, doi:10.1016/j.tig.2010.05.003 (2010).
21 Ossowski, S. *et al.* The rate and molecular spectrum of spontaneous mutations in Arabidopsis thaliana. *Science* **327**, 92-94, doi:10.1126/science.1180677 (2010).
22 Exposito-Alonso, M. *et al.* The rate and potential relevance of new mutations in a colonizing plant lineage. *PLoS Genet* **14**, e1007155, doi:10.1371/journal.pgen.1007155 (2018).
23 Bartley, B. G. D. *The genetic diversity of cacao and its utilization*. (CABI Publishing, 2005).
24 Kidd, J. M. *et al.* Population genetic inference from personal genome data: impact of ancestry and admixture on human genomic variation. *Am J Hum Genet* **91**, 660-671, doi:10.1016/j.ajhg.2012.08.025 (2012).
25 Terhorst, J., Kamm, J. A. & Song, Y. S. Robust and scalable inference of population history from hundreds of unphased whole genomes. *Nature Genetics* **49**, 303-309, doi:10.1038/ng.3748 (2017).
26 Gutenkunst, R. N., Hernandez, R. D., Williamson, S. H. & Bustamante, C. D. Inferring the joint demographic history of multiple populations from multidimensional SNP frequency data. *PLoS Genet* **5**, e1000695, doi:10.1371/journal.pgen.1000695 (2009).
27 Wright, S. Coefficients of inbreeding and relationship. *American Naturalist* **56**, 330–338 (1922).
28 Crow, J. F. & Kimura, M. *An Introduction to Population Genetics Theory*. 591 (Blackburn Press, 1970).
29 Cope, F. W. The mechanism of pollen incompatibility in *Theobroma cacao* L. *Heredity* **17**, 157-182 (1962).
30 Lanaud, C. *et al.* Deciphering the Theobroma cacao self-incompatibility system: from genomics to diagnostic markers for self-compatibility. *J Exp Bot* **68**, 4775-4790, doi:10.1093/jxb/erx293 (2017).
31 Nordborg, M. Linkage disequilibrium, gene trees and selfing: an ancestral recombination graph with partial selffertilization.
Genetics **154**, 923–929 (2000).
32 Nordborg, M. & Donnelly, P. The coalescent process with selfing. *Genetics* **146**, 1185–1195 (1997).
33 Motamayor, J. C. *et al.* Cacao domestication I: the origin of the cacao cultivated by the Mayas. *Heredity (Edinb)* **89**, 380-386, doi:10.1038/sj.hdy.6800156 (2002).
34 Henderson, J. S., Joyce, R. A., Hall, G. R., Hurst, W. J. & McGovern, P. E. Chemical and archaeological evidence for the earliest cacao beverages. *P Natl Acad Sci USA* **104**, 18937-18940, doi:10.1073/pnas.0708815104 (2007).

35 Powis, T. G., Cyphers, A., Gaikwad, N. W., Grivetti, L. & Cheong, K. Cacao use and the San Lorenzo Olmec. *P Natl Acad Sci USA* **108**, 8595-8600, doi:10.1073/pnas.1100620108 (2011).
36 Schultes, R. E. in *Pre-Columbian plant migration, Papers of the Peabody Museum of Archaeology and Ethnology* Vol. 76 (ed D. Stone) 69–83 (Harvard University Press, 1984).
37 Loor Solorzano, R. G. *et al.* Insight into the wild origin, migration and domestication history of the fine flavour Nacional Theobroma cacao L. variety from Ecuador. *PLoS One* **7**, e48438, doi:10.1371/journal.pone.0048438 (2012).
38 Abecasis, G. R. *et al.* Extent and distribution of linkage disequilibrium in three genomic regions. *Am J Hum Genet* **68**, 191-197, doi:10.1086/316944 (2001).
39 Chen, H., Patterson, N. & Reich, D. Population differentiation as a test for selective sweeps. *Genome Res* **20**, 393-402, doi:10.1101/gr.100545.109 (2010).
40 Vaser, R., Adusumalli, S., Leng, S. N., Sikic, M. & Ng, P. C. SIFT missense predictions for genomes. *Nat Protoc* **11**, 1-9, doi:10.1038/nprot.2015.123 (2016).
41 Lu, J. *et al.* The accumulation of deleterious mutations in rice genomes: a hypothesis on the cost of domestication. *Trends Genet* **22**, 126-131, doi:10.1016/j.tig.2006.01.004 (2006).
42 Renaut, S. & Rieseberg, L. H. The Accumulation of Deleterious Mutations as a Consequence of Domestication and Improvement in Sunflowers and Other Compositae Crops. *Molecular Biology and Evolution* **32**, 2273-2283, doi:10.1093/molbev/msv106 (2015).
43 Mezmouk, S. & Ross-Ibarra, J. The pattern and distribution of deleterious mutations in maize. *G3 (Bethesda)* **4**, 163-171, doi:10.1534/g3.113.008870 (2014).
44 Livingstone, D., 3rd *et al.* A Larger Chocolate Chip-Development of a 15K Theobroma cacao L. SNP Array to Create High-Density Linkage Maps. *Front Plant Sci* **8**, 2008, doi:10.3389/fpls.2017.02008 (2017).
45 Danecek, P. *et al.* The variant call format and VCFtools. *Bioinformatics* **27**, 2156-2158, doi:10.1093/bioinformatics/btr330 (2011).
46 Bekele, F. L., Bekele, I., Butler, D. R. & Bidaisee, G. G. Patterns of morphological variation in a sample of cacao (Theobroma cacao L.) germplasm from the International Cocoa Genebank, Trinidad. *Genetic Resources and Crop Evolution* **53**, 933-948 (2006).
47 Ciferri, R. & Ciferri, F. The evolution of Cultivated cacao. *Evolution* **11**, 381-397 (1957).
