# Supplementary material for: Population genomic analyses of the chocolate tree, Theobroma cacao L., provide insights into its domestication process
Source: Commun Biol. 2018 Oct 16;1:167. doi: 10.1038/s42003-018-0168-6 (PMC6191438; doi:10.1038/s42003-018-0168-6)
Supplement: Supplementary file 2 — Description of additional supplementary items [file 42003_2018_168_MOESM2_ESM.docx]

**Description of additional supplementary items**

**Supplementary Data 1: Genes within regions of the genome identified under directional selection in Criollo, when compared to Curaray.** Only genes found within windows that show significant signatures of selection at p < 0.005 were considered.

**Supplementary Data 2: Putative Genes within regions of the genome identified as Fst outliers between Criollo and Curaray populations.**
